# The Burden of Type 1 and Type 2 Diabetes Among Adolescents and Young Adults in 24 Western European Countries, 1990–2019: Results From the Global Burden of Disease Study 2019

**DOI:** 10.3389/ijph.2023.1606491

**Published:** 2024-02-14

**Authors:** Benedetta Armocida, Lorenzo Monasta, Susan M. Sawyer, Flavia Bustreo, Graziano Onder, Giulio Castelpietra, Flavia Pricci, Valentina Minardi, Claudia Giacomozzi, Cristiana Abbafati, Lauryn K. Stafford, Maja Pasovic, Simon I. Hay, Kanyin Lian Ong, Pablo Perel, David Beran, GBD 2019 Europe Adolescent Diabetes

**Affiliations:** ^1^ Division of Tropical and Humanitarian Medicine, University of Geneva, Geneva, Switzerland; ^2^ Department of Cardiovascular, Endocrine-Metabolic Diseases and Aging, Istituto Superiore di Sanità, Rome, Italy; ^3^ Institute for Maternal and Child Health—IRCCS Burlo Garofolo, Trieste, Italy; ^4^ Department of Paediatrics, Murdoch Children’s Research Institute, Centre for Adolescent Health, Royal Children’s Hospital Melbourne, University of Melbourne, Melbourne, VIC, Australia; ^5^ Fondation Botnar, Geneva, Switzerland; ^6^ Fondazione Policlinico Gemelli IRCCS, Rome, Italy; ^7^ Department of Geriatric and Orthopedic Sciences, Università Cattolica del Sacro Cuore, Rome, Italy; ^8^ Outpatient and Inpatient Care Service, Central Health Directorate, Trieste, Italy; ^9^ National Centre for Disease Prevention and Health Promotion, Istituto Superiore di Sanità, Rome, Italy; ^10^ Department of Juridical and Economic Studies, Sapienza University of Rome, Rome, Italy; ^11^ Institute for Health Metrics and Evaluation, Seattle, WA, United States; ^12^ Department of Health Metrics Sciences, School of Medicine, University of Washington, Seattle, WA, United States; ^13^ Department of Non-Communicable Disease Epidemiology, London School of Hygiene and Tropical Medicine, London, United Kingdom; ^14^ Division of Tropical and Humanitarian Medicine, University of Geneva and Geneva University Hospitals, Geneva, Switzerland

**Keywords:** adolescent health, diabetes, global burden of disease, Western Europe, public health

## Abstract

**Objectives:** As little is known about the burden of type 1 (T1DM) and type 2 diabetes (T2DM) in adolescents in Western Europe (WE), we aimed to explore their epidemiology among 10–24 year-olds.

**Methods:** Estimates were retrieved from the Global Burden of Diseases Study (GBD) 2019. We reported counts, rates per 100,000 population, and percentage changes from 1990 to 2019 for prevalence, incidence and years lived with disability (YLDs) of T1DM and T2DM, and the burden of T2DM in YLDs attributable to high body mass index (HBMI), for 24 WE countries.

**Results:** In 2019, prevalence and disability estimates were higher for T1DM than T2DM among 10–24 years old adolescents in WE. However, T2DM showed a greater increase in prevalence and disability than T1DM in the 30 years observation period in all WE countries. Prevalence increased with age, while only minor differences were observed between sexes.

**Conclusion:** Our findings highlight the substantial burden posed by DM in WE among adolescents. Health system responses are needed for transition services, data collection systems, education, and obesity prevention.

## Introduction

Diabetes mellitus (DM) is a major global public health issue as it imposes a heavy burden on mortality and disability, as well as on socio-economic development [1]. DM is one of five priority non-communicable diseases (NCDs) targeted in the 2011 Political Declaration on the Prevention and Control of NCDs [2]. The Sustainable Development Goals (SDGs) also include a target to reduce the proportion of premature deaths due to NCDs, including DM, by one third by 2030, with new commitments made at the 74th World Health Assembly [3] to strengthen efforts around prevention and control, as well as a set of global targets for diabetes coverage. In 2021, approximately 537 million people had diabetes worldwide, a number which is estimated to rise to 700 million by 2045 [4]. Type 1 Diabetes Mellitus (T1DM) is one of the most common endocrine and metabolic conditions in childhood and adolescence. The European Region has the highest number of children and adolescents living with T1DM compared to any other global region, reaching about 295,000 in total [4]. Wide variation in incidence of T1DM in children younger than 15 years is described by registry reports from the EURODIAB study group within Europe [5], which also indicated an overall pooled rate of annual increases of 3.4% (95% Confidence interval 2.8%–3.9%).

Until recently, type 2 Diabetes Mellitus (T2DM) was referred to as “adult-onset diabetes,” reflecting it as a disorder of ageing. Yet its prevalence is also increasing in children and adolescents, albeit highly variable among countries. According to the literature, the incidence of T2DM between the ages of 10 and 19 years in the United States was 12.5 cases per 100,000 in 2011–2012, while in the United Kingdom and other European countries, its incidence has been much lower, at <1 case per 100,000 [6], although higher in specific ethnic minorities. In 2018, across the European Union (EU), almost one in five 15 year-olds was either overweight or obese [7]. The rise in prevalence of overweight or obesity [8], which are major risk factors for T2DM, is thought to have contributed to rising rates of T2DM in adolescents [9]. Indeed, screening studies have reported the prevalence of T2DM of 0.4% up to 1% in obese adolescents ≥12 years [10]. The rise of T2DM among adolescents is a major public health concern, as earlier onset of DM implies a longer lifetime exposure to hyperglycaemia, and a consequent larger predisposition to long-term complications (e.g., renal failure, cardiovascular morbidity, retinopathy, neuropathy), premature death, and poor quality of life [11].

To our knowledge, despite the available population studies, no comprehensive analysis is yet available in Europe on both types of DM, and reliable data on adolescents are still sparse [12]. Indeed, considering their effects on health and quality of life, and the substantial direct and indirect economic burden [12], investigation of the burden of T1DM and T2DM in adolescents in European countries is overdue. In this regard, Western Europe is an important sub-region to explore the burden and epidemiological trends of T1DM and T2DM among adolescents, given the considerable epidemiological similarities and homogeneity among countries. Given the inconsistency and sparsity in data collection systems and the major data gaps for adolescents [4], we used estimates provided by the Global Burden of Diseases, Injuries, and Risk Factors study 2019 (GBD 2019) to: 1) describe the prevalence, incidence, and disability burden of T1DM and T2DM among 10–24 year old adolescents by age, sex, and country in 2019; 2) assess the disease burden of T1DM and T2DM through temporal trends using prevalence, incidence and years lived with disability (YLDs), from 1990 to 2019 at sub-regional level and national level; 3) estimate the burden of T2DM in YLDs attributable to high body mass index (HBMI).

## Methods

This study adopted the broad age definition for adolescence from 10 to 24 years because it accurately captures the biological, social, and neurocognitive development of this population [13]. Additionally, it used the GBD 2019 country grouping for Western Europe, which comprised 24 countries, including Israel, that were grouped based on epidemiological similarities and/or geographical proximity.

### GBD Overview

The GBD 2019 generated estimates using 86,249 sources, and produced estimates of incidence, prevalence, mortality, YLD, Years of Life Lost (YLLs), Disability-adjusted life years (DALYs), life expectancy, and health-adjusted life expectancy. It complies with the Guidelines for Accurate and Transparent Health Estimates Reporting statement [14]. For most diseases and injuries, data are modelled using three main standardised tools [Cause of Death Ensemble model (CODEm), spatiotemporal Gaussian process regression (ST-GPR), and Disease Modelling-Meta regression (DisMod-MR)] to produce estimates of each quantity of interest by age, sex, location, and year [15], accompanied by the relative 95% Uncertainty Intervals (UIs). These represent the 25th and 975th ordered estimates of 1,000 draw estimates of the posterior distribution [15]. Methods for the GBD 2019 estimates are described in detail in the capstone papers and appendices [15, 16]. The GBD 2019 cycle provides a complete set of comparable health measures for 204 countries, with a comprehensive and systematic analysis of 286 causes of death, 369 causes of disease and injury, and 87 risks factors. The GBD 2019 cause-list is composed of a four-level hierarchy, with each level comprising mutually exclusive and collectively exhaustive causes. T1DM and T2DM are included in the level 4 hierarchy under the non-communicable diseases (NCDs) level 1 group. Their definitions are reported in capstones papers, appendices [15, 16] and can be publicly retrieved at [17]. GBD 2019 assumes that all DM under 15 years of age is T1DM. The estimates are modelled based on systematic reviews of published and unpublished documents, survey microdata, administrative records of health encounters, diabetes registries, and disease surveillance systems whose references are freely available on the Global Health Data Exchange website [18]. For non-fatal health outcome related to DM in Western Europe, the Data Input Sources Tool reports 30,303 total source metadata rows from 303 citations.

### Data Analysis

T1DM and T2DM estimates for incidence, prevalence, YLDs were extracted from the Global Health Data Exchange website (http://ghdx.healthdata.org). YLDs is a metric estimated by multiplying the prevalence counts with the disability weight for a given disease or injury; for T1DM and T2DM, YLDs are estimated by assigning disability weights to specific health states within the T1DM and T2DM conditions, proportionally to their prevalence [15, 16]. As the age (10–24 years old) and location (Western Europe) of the study population mortality rate estimates was 0.09 per 100,000 population, we primarily focused on non-fatal burden. Estimates were retrieved for 24 Western European countries, from 1990 to 2019, for 10–24 year-olds, as this expanded age definition may facilitate extended investments in different settings.

Estimates, accompanied by 95% UI, were extracted for males and females separately and jointly, and for three age groups [10–14 years (younger adolescents); 15–19 years (older adolescents); 20–24 years (young adults)] [14]. The Western European countries included were: Andorra, Austria, Belgium, Cyprus, Denmark, Finland, France, Germany, Greece, Iceland, Ireland, Israel, Italy, Luxembourg, Malta, Monaco, Netherlands, Norway, Portugal, San Marino, Spain, Sweden, Switzerland, and the United Kingdom (UK). We analysed prevalence, incidence and YLD rates for T1DM and T2DM, by time (1990–2019), country, age-groups, and sex. We also reported the proportion of T2DM YLD rate attributed to HBMI, by sex, 30 years period and country. As the population attributable fraction (PAF) for risk factor and disease pairs are exclusively calculated for ages where both the risk and the disease are estimated, the PAF attributed to HBMI has only been calculated for 20–24 year-olds [16]. We considered estimates to be significantly different by determining whether the 95% UIs overlapped. Data were analysed and visualisations prepared in Tableau 2021.4.16.

## Results

In 2019, there were 320,237 (95% UI 234,473–408,257) adolescents aged 10–24 years old in the 24 Western European countries with T1DM, and 227,299 (130,968–345,987) with T2DM. Age groups disaggregation are reported in Supplementary Tables S1, S2.

### Prevalence of T1DM and T2DM

In 2019, as shown in Supplementary Figure S1, T1DM was more prevalent in adolescents than T2DM; T1DM accounted for 447.4 per 100,000 population (327.6–570.3) while T2DM accounted for 317.5 (183.0–483.3) (Supplementary Table S2). In 2019, an increase in prevalence rates was observed in relation to age, but this was significantly higher in T2DM than T1DM [T2DM: 141.7 (35.8–303.0) 15–19 years old; 791.1 (480.9–1143.8) 20–24 years old] (Supplementary Figure S2). No differences were observed by sex (Supplementary Table S2; Supplementary Figure S2).

**FIGURE 1:**
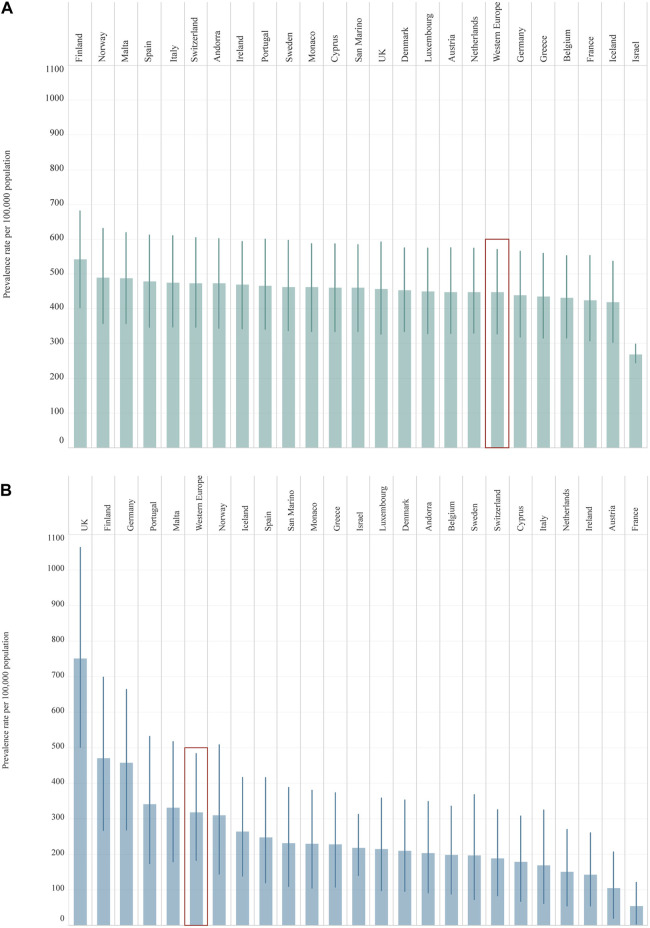
Prevalence rate per 100,000 population (uncertainty intervals), both sexes, 10–24 years old, in 2019, by country: Type 1 Diabetes Mellitus (Panel **(A)**); Type 2 Diabetes Mellitus (Panel **(B)**). Abbreviations: T1DM, diabetes mellitus type 1; T2DM, diabetes mellitus type 2; UK, United Kingdom. Legend: T1DM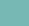 T2DM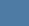 (Global Burden of Disease Study, 24 Western Europe countries, 2019).

From 1990 to 2019, a greater increase was seen in the prevalence rate of T2DM [122.9% (90.8–177.5)] than of T1DM, although this has also increased over the past 30 years [57.0% (40.1–72.4)] (Table 1). In 2019, Finland was the country with the highest prevalence rate of T1DM in adolescents [542.7 (402.9–681.0)] while Israel had the lowest prevalence [268.6 (244.6–298.1)] (Table 1; Figure 1). In two countries (Cyprus and France), an increase in percentage change of more than 100% was observed for the prevalence rate of T1DM from 1990 to 2019. For T2DM, eight countries had a percentage change increased in prevalence of more than 100% over this period. Finland and Israel showed a decrease in the percentage change for T1DM [−19.0% (−39.4 to 1.2) and −4.2 (−25.5 to 34.2) respectively] from 1990 to 2019, and a steady increase of more than 200% for T2DM, while Sweden had more modest changes in prevalence rate for both T1DM and T2DM (Table 1).

**TABLE 1:** Type 1 Diabetes Mellitus and Type 2 Diabetes Mellitus prevalence rate per 100,000 population (uncertainty intervals), in adolescents aged 10–24 years, in Western Europe and by countries, in 1990 and 2019, and percentage of change from 1990 to 2019 (uncertainty intervals) (Global Burden of Disease Study, 24 Western Europe countries, 1990–2019).

Location	T1DM						Percentage change (1990–2019) (%)			T2DM						Percentage change (1990–2019) (%)		
	1990			2019						1990			2019					
	Rate	95% UI		Rate	95% UI		%	95% UI		Rate	95% UI		Rate	95% UI		%	95% UI	
Western Europe	288.9	230.8	353.8	447.4	327.6	570.3	57.0	40.1%	72.4%	144.4	75.9	229.0	317.5	183.0	483.3	122.9	90.8%	177.5%
Andorra	295.9	212.8	397.1	473.5	344.1	601.4	61.1	36.0%	75.9%	126.6	40.2	235.0	203.7	91.6	348.5	61.9	18.1%	191.8%
Austria	237.7	183.6	306.4	448.5	329.0	575.1	89.8	57.7%	123.3%	58.4	14.4	107.8	104.3	19.9	206.6	79.6	−28.2%	230.8%
Belgium	256.3	192.1	338.2	431.8	315.9	552.5	69.5	44.4%	90.5%	117.2	46.0	202.6	198.8	88.2	335.4	70.6	18.6%	157.3%
Cyprus	229.0	185.3	286.0	459.7	334.3	586.4	100.9	65.0%	135.5%	88.4	41.4	142.7	178.4	67.8	307.6	101.8	19.3%	197.7%
Denmark	289.1	215.6	387.5	452.9	334.3	574.9	57.6	35.4%	76.8%	128.1	47.9	226.7	210.0	95.7	352.8	64.9	18.2%	166.2%
Finland	674.0	647.9	701.9	542.7	402.9	681.0	−19.0	−39.4%	1.2%	123.0	47.2	225.8	471.0	267.4	698.5	285.4	163.6%	624.7%
France	185.8	157.2	221.5	424.7	307.6	552.6	134.5	86.6%	182.1%	35.3	12.3	61.1	54.6	3.7	121.0	58.6	−78.8%	186.0%
Germany	245.8	194.6	303.8	438.8	318.8	564.9	80.5	43.4%	123.8%	275.6	174.1	389.9	456.8	268.5	664.0	67.5	26.4%	108.9%
Greece	225.4	174.3	292.6	435.6	315.7	559.2	99.5	66.8%	132.5%	96.0	41.4	169.1	227.5	108.0	373.2	144.6	63.9%	266.0%
Iceland	264.6	190.0	355.9	418.4	303.5	536.3	59.5	34.7%	75.4%	119.6	43.1	209.8	263.4	139.0	416.1	122.0	68.4%	302.9%
Ireland	255.1	205.7	315.4	469.4	342.4	593.1	85.5	55.9%	115.3%	93.0	29.8	167.0	142.2	54.7	260.4	54.1	−6.3%	176.7%
Israel	284.0	197.7	378.6	268.6	244.6	298.1	−4.2	−25.5%	34.2%	70.9	20.7	146.1	217.6	140.4	312.4	210.7	62.2%	757.2%
Italy	409.7	297.4	529.6	475.0	347.6	609.8	17.2	8.8%	24.4%	84.8	19.8	190.4	169.4	61.9	324.6	102.0	60.3%	264.7%
Luxembourg	275.8	205.0	363.4	449.0	328.4	574.3	64.3	43.1%	82.0%	152.5	68.1	262.6	215.5	97.8	358.2	42.7	−2.5%	109.0%
Malta	296.9	215.2	398.4	488.3	357.6	618.6	64.1	40.1%	80.5%	235.5	123.8	362.3	331.9	179.1	517.1	40.6	5.2%	90.7%
Monaco	302.9	220.1	399.5	461.7	334.5	586.7	53.5	31.0%	67.5%	146.0	52.8	257.5	229.2	104.9	380.1	58.1	20.1%	172.5%
Netherlands	274.7	211.6	354.9	447.6	330.0	574.1	63.4	41.1%	86.9%	85.3	27.0	160.9	151.4	54.7	270.1	78.1	8.6%	231.0%
Norway	425.7	309.8	541.8	489.5	357.6	630.9	15.0	4.3%	24.4%	232.3	95.7	406.6	309.7	144.5	508.2	33.3	15.9%	63.5%
Portugal	270.3	204.0	353.1	466.2	341.2	599.9	73.0	47.9%	98.0%	220.4	117.3	341.5	340.9	173.8	531.8	55.1	9.5%	114.0%
San Marino	291.9	210.2	389.9	459.4	334.3	584.0	58.0	34.5%	73.1%	133.4	44.1	245.0	230.9	109.5	388.0	73.7	27.1%	207.9%
Spain	277.8	243.0	315.6	478.9	346.9	611.6	75.9	37.2%	112.5%	93.1	46.1	152.2	247.9	119.7	415.8	171.6	83.4%	318.2%
Sweden	456.7	337.0	584.9	462.5	336.9	596.5	2.5	−3.8%	9.5%	148.9	32.5	302.3	196.1	72.6	367.8	33.3	−3.4%	162.9%
Switzerland	300.8	218.7	405.1	473.6	347.0	604.2	58.9	35.1%	72.1%	115.0	27.4	216.8	187.9	83.4	325.4	64.9	19.1%	230.0%
United Kingdom	305.3	232.4	392.0	456.3	327.0	591.9	50.8	31.0%	67.5%	239.5	116.6	391.3	751.0	501.2	1,063.3	216.4	151.4%	371.3%

Abbreviation: T1DM, Type 1 diabetes mellitus; T2DM, Type 2 diabetes mellitus; 95% UI, 95% uncertainty interval.

### Incidence of T1DM and T2DM

In 2019, T2DM incidence rates per 100,000 population among 10–24 years old in Western Europe was 3.5 times higher than T1DM [87.2 (60.3–120.2) T2DM; 24.9 (16.7–34.4) T1DM] (Supplementary Table S2). Differences among age groups and sex are reported in Supplementary Table S2 and Supplementary Figure S3. In 2019, there was a higher incidence of T1DM among 10–14 year-olds than older age groups. While it was slightly higher in males, sex differences were minor for T1DM. Although 15–19 year-old females had a higher incidence rate than males for T2DM, overall, the highest incidence was observed in 20–24 year-olds and in males (Supplementary Figure S3). Marked variability in incidence rate by country was observed in T2DM with a 5-fold difference in incidence rate between the highest {UK [180.2 (129.2–243.0)]} and the lowest country {France [36.0 (22.1–53.1)]} (Supplementary Table S3). In contrast, there was less variability between Western European countries around the incidence rate of T1DM, with around a 2-fold difference between countries having the highest {Finland [32.6 (22.4–44.6)]} and lowest incidence {Israel [16.6 (14.7–18.8)]} (Supplementary Table S3 and Supplementary Figure S4). In all 24 countries, there was a greater incidence of T2DM than T1DM (Supplementary Figure S4). For T2DM, there was a significantly higher incidence rate in the UK compared to the Western Europe sub-region; the lowest incidence was in France. From 1990 to 2019 the incidence rate of T2DM increased by 158.3% (134.6–191.0), while for T1DM it increased by 75.5% (47.3–100.5) (Supplementary Table S3). Over the past 30 years, six countries (Austria, Cyprus, France, Germany, Greece, Portugal) had a percentage change in the incidence of T1DM of more than 100%; half of these countries had a change of more than 150%. Finland was the only country where a reduction in incidence of T1DM was observed over this period [−7.5% (−35.8 to 25.3)] (Supplementary Table S3). For T2DM, 21 countries had a percentage change in incidence of more than 100% over the past 30 years. The lowest percentage change was observed in Norway [42.2% (34.9–54.8)] (Supplementary Table S3).

### Years Lived With Disabilities for T1DM and T2DM

**FIGURE 2:**
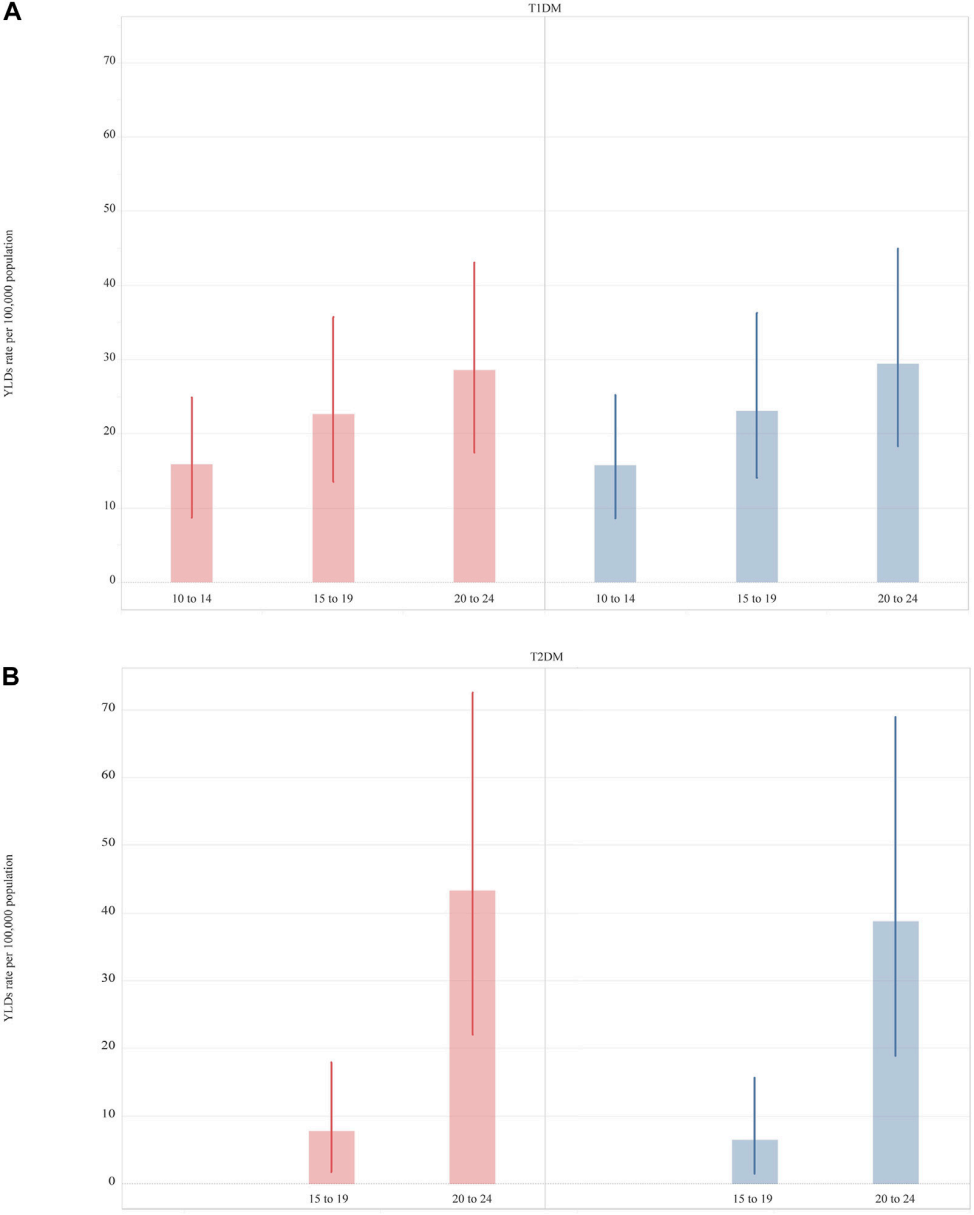
Years lived with disability rate per 100,000 (uncertainty intervals) of Type 1 Diabetes Mellitus and Type 2 Diabetes Mellitus, in 2019, by sex and age. Type 1 Diabetes Mellitus (Panel **A**); Type 1 Diabetes Mellitus (Panel **B**). (Global Burden of Disease Study, Western Europe region, 2019).

In 2019, T1DM imposed a slightly higher YLD rate [22.7 (13.7–35.0)] among adolescents than T2DM [16.4 (7.9–28.5)]. In 2019, while an increase with age of YLDs was observed in both T1DM and T2DM, differences by sex in YLD rates were minor (Figure 2). For T2DM, YLDs rate was slightly higher in 15–19 year-olds and 20–24 year-old females and significantly different in the two age groups (Supplementary Table S2 and Figure 2). Apart from the United Kingdom and Germany, Western European countries had a higher rate of YLDs for T1DM than for T2DM (Supplementary Figure S5). The highest YLD rates for T1DM were in Finland and the lowest in Israel (Table 2). For T2DM, the highest YLD rate was reported in the UK and Finland and the lowest in France (Supplementary Figure S5 and Table 2). From 1990 to 2019, YLD rates for T1DM had increased by 56.3% (38.7–72.8). The greatest increases in YLD rates for T1DM were observed in France [135.9% (80.8–200.7)], while the lowest were in Finland [−18.7% (-39.5 to 3.6)] (Table 2). For T2DM, a greater change over the 30 year period was observed [123.0% (88.9–181.4)]. Seven countries (Finland, Greece, Iceland, Israel, Italy, Spain, and UK) experienced a percentage change of more than 100%, of which three (Finland, Israel, and UK) reported a percentage change of more than 200% (Table 2).

**TABLE 2:** Type 1 Diabetes Mellitus and Type 2 Diabetes Mellitus years lived with disability rate per 100,000 population (uncertainty intervals), in adolescents aged 10–24 years, in Western Europe and by countries, in 1990 and 2019, and percentage of change from 1990 to 2019 (uncertainty intervals) (Global Burden of Disease Study, 24 Western Europe countries, 1990–2019).

Location	T1DM						Percentage of change (%)			T2DM						Percentage of change (%)		
	1990			2019						1990			2019					
	Rate	95% UI		Rate	95% UI		%	95% UI		Rate	95% UI		Rate	95% UI		%	95% UI	
Western Europe	14.7	9.2	22.2	22.7	13.7	35.0	56.3	38.7%	72.8%	7.5	3.3	13.6	16.4	7.9	28.5	123.0	88.9%	181.4%
Andorra	15.1	9.0	24.1	24.1	14.4	38.0	60.9	31.0%	92.1%	6.6	1.7	13.9	10.7	4.1	21.0	63.4	10.2%	214.2%
Austria	12.4	7.3	19.0	23.4	14.1	36.1	90.3	50.0%	138.1%	3.1	0.7	6.8	5.6	1.0	12.5	80.7	−27.2%	284.1%
Belgium	13.4	7.9	21.6	22.6	13.7	35.5	66.5	34.2%	101.6%	6.4	2.3	12.7	10.8	4.2	21.2	68.6	12.3%	175.9%
Cyprus	11.7	7.2	17.6	23.5	14.3	36.3	95.9	52.1%	143.6%	4.6	1.8	8.9	9.3	3.0	18.9	95.4	12.8%	214.5%
Denmark	14.5	8.7	22.6	22.6	13.4	34.9	58.4	30.6%	90.7%	6.5	2.1	13.1	10.7	4.1	20.6	67.6	14.5%	183.0%
Finland	34.4	22.3	50.2	27.6	16.9	42.4	−18.7	−39.5%	3.6%	6.4	2.0	13.0	24.5	11.9	42.3	285.5	148.6%	680.1%
France	9.3	5.8	14.3	21.2	12.6	33.0	135.9	80.8%	200.7%	1.8	0.5	3.6	2.8	0.2	7.5	61.5	−77.9%	241.3%
Germany	12.4	7.7	19.2	22.0	12.9	34.5	77.8	33.9%	131.9%	14.0	7.3	23.6	23.4	11.8	39.9	66.2	20.3%	119.9%
Greece	11.7	7.1	18.0	22.6	13.5	35.1	90.4	48.7%	138.7%	5.2	1.9	10.4	12.3	4.6	23.3	135.0	51.2%	288.6%
Iceland	13.5	7.9	21.3	21.3	12.6	33.2	57.0	27.6%	88.8%	6.2	1.9	12.1	13.8	6.0	24.8	120.4	53.7%	327.4%
Ireland	13.0	8.1	19.5	23.8	14.2	37.5	78.4	42.6%	119.4%	4.8	1.3	9.7	7.4	2.5	15.3	49.4	−11.6%	185.3%
Israel	14.4	8.1	22.5	13.7	8.8	20.4	−4.8	−29.4%	36.2%	3.7	0.9	8.5	11.4	6.0	19.2	203.7	54.2%	798.7%
Italy	21.3	13.1	33.2	24.2	14.8	37.7	18.5	8.5%	27.4%	4.5	1.0	11.1	8.8	2.9	18.6	104.0	60.0%	273.7%
Luxembourg	14.0	8.4	21.7	22.9	13.6	35.0	68.0	35.6%	104.7%	7.9	2.8	15.3	11.3	4.3	21.4	47.3	−3.8%	133.4%
Malta	15.1	8.9	24.3	24.9	14.9	38.7	57.2	28.2%	88.2%	12.2	5.5	21.5	17.4	7.9	32.3	35.0	−3.7%	89.3%
Monaco	15.5	9.3	24.2	23.5	14.2	36.1	53.8	24.7%	81.6%	7.6	2.2	15.7	11.9	4.7	22.3	59.4	13.6%	175.7%
Netherlands	14.1	8.6	21.7	23.1	14.0	35.9	61.3	29.2%	99.6%	4.5	1.2	9.8	8.1	2.5	16.2	76.3	−2.0%	237.3%
Norway	21.6	13.1	33.2	24.8	15.1	38.7	13.6	1.7%	25.2%	12.0	4.5	23.6	16.0	6.7	29.7	32.0	13.3%	65.9%
Portugal	13.7	8.2	21.5	23.7	14.4	36.4	76.6	41.5%	114.1%	11.4	5.0	20.3	17.8	8.0	31.5	59.5	9.8%	122.8%
San Marino	14.9	8.8	23.5	23.4	14.2	36.0	56.7	28.6%	86.3%	6.9	2.0	14.8	12.1	4.8	23.1	73.8	17.5%	245.2%
Spain	14.4	9.2	21.4	24.8	14.6	38.5	70.0	27.6%	115.4%	5.0	1.9	9.8	13.3	5.5	25.2	163.7	64.4%	342.8%
Sweden	22.9	13.7	35.4	23.3	14.1	35.6	2.0	−10.0%	15.2%	7.6	1.4	17.5	10.0	3.2	21.2	32.0	−8.1%	177.2%
Switzerland	15.3	9.2	24.1	24.1	14.5	37.3	73.9	43.7%	110.3%	6.0	1.2	13.1	9.8	3.4	19.3	81.0	16.3%	301.7%
United Kingdom	15.3	9.5	23.9	22.8	13.8	35.1	54.7	33.6%	72.5%	12.2	5.1	22.0	38.1	20.7	62.8	224.9	157.9%	383.6%

Abbreviation: T1DM, Type 1 diabetes mellitus; T2DM, Type 2 diabetes mellitus; YLDs, Years Lived with Disabilities; 95% UI, 95% uncertainty interval.

### YLDs Attributed Risk Factors

**FIGURE 3:**
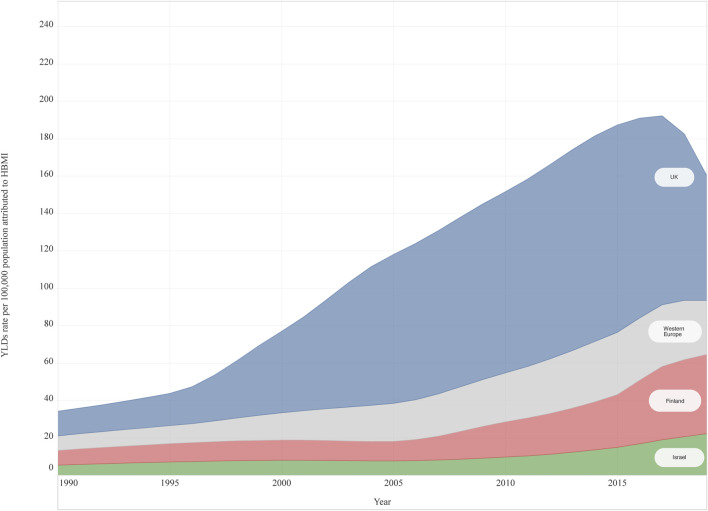
High body mass Index attributable years lived with disability rate per 100,000 for Type 2 Diabetes Mellitus, both sexes, 20–24 years old, from 1990 to 2019, in three countries and Western Europe (Global Burden of Disease Study, United Kingdom, Finland, Israel and Western Europe Region, 1990–2019). Abbreviations: HBMI, high body mass index; YLDs, Years lived with disability; UK, United Kingdom *Note: The countries were selected for being the top three countries having the highest percentage change (1990–2019).

In 2019, HBMI accounted for 28.8 (12.6–54.9) of the attributable YLD rates per 100,000 population for T2DM among adolescents aged 20–24 years in Western Europe. No differences were observed by sex in Western Europe, but the YLD rate was slightly higher in females [female 31.0 (13.6–58.8); male 26.8 (11.6–52.7)]. Variation among countries was observed. The UK had the highest attributable YLD rate for HBMI 66.8 (32.4–117.5) and France had the lowest at 6.6 (0.4–17.3). UK, Finland, and Germany reported a higher YLD rate compared to the Western Europe sub-region. Significant differences among countries were observed between UK (highest) and France, Austria, Cyprus, and Italy (Figure 3). All countries, except for UK, Norway and Italy reported higher YLDs rate attributed to HBMI among females (Supplementary Figure S6). From 1990 to 2019, an increase in YLDs attributable to HBMI of 271.9% (180.6–536.8) was observed in Western Europe among 20–24 year-olds and in both sexes (Figure 3), with the highest increase reported in Finland [435.6% (218.3–1382.6)]. Only one country (Norway) had increased less than 100% (Supplementary Table S4).

## Discussion

This study is the first systematic analysis of the incidence, prevalence, and disability burden of both types of DM among adolescents in Western Europe using GBD 2019 estimates. Overall, our findings highlight the increase in incidence, prevalence, and disability burden of T1DM and T2DM. All 24 countries reported a rising trend of the three measures in T2DM over the 30 years from 1990 to 2019. This was very similar for T1DM, in that 22 of 24 countries reported increases in prevalence and YLD rates, and 23 countries reported an increase in incidence. While T1DM remains the predominant type of DM in adolescents in Western Europe, there is a striking increase in the incidence of T2DM [19], showing that this disease should not only be considered an adult-onset disease. At the very least, one of the reasons for an expected increase in prevalence of T1DM is the decline in mortality that accompanies higher quality healthcare resulting in greater survival into adolescence from incident T1DM in earlier childhood.

The rising prevalence of adolescent DM also presents new challenges for health systems. Innovative approaches are needed as the best outcomes for both types of DM require high levels of self-care by adolescents which is best supported by patient-centred care and multi-disciplinary models of care that span primary and specialist settings. A paradigm shift is required for many paediatric diabetes services which predominantly focus on T1DM and are dominated by technological advances that are less relevant for those with T2DM. Most older adolescents will be managed by adult-oriented health services. While these are typically more concerned in addressing T2DM, they are also historically oriented to older adults than adolescents. Puberty is associated with metabolic changes that of itself are thought to be associated with greater insulin resistance [20]. These biological changes intersect with the wider aspects of psychosocial development that define adolescence, with poorer adherence to treatment, initiation of risky behaviours, incident eating and mental health disorders, which are associated with complications of DM [20]. Greater self-care is required by adolescents, which is supported by carers who are equally responsible for shaping their children’s health behaviours and supervising medical management. Negotiating these changing roles and responsibilities can impose considerable challenges for individuals and their families, as well as healthcare teams [20]. Given that the transfer from paediatric to adult services typically occurs between 15 and 19 years of age [21], a further challenge for diabetes care results from the challenge of transferring specialist healthcare from paediatric oriented to adult providers [20]. Improving the health-related quality of life for adolescents is an important priority during the transition from paediatric to adult healthcare [22], which beyond the medical supervision of insulin regimens requires attention to diabetes education, social support, and psychological services. Educating and training the current and future health workforce about how to work with adolescent patients and their families is globally indicated [21], as well as in Europe [23].

In line with other studies, our findings also highlight the higher prevalence of T2DM and higher HBMI attributable YLD for T2DM among female adolescents. These sex differences might be linked to differential sex-hormone effects, undiagnosed polycystic ovary syndrome, divergent post pubertal weight gain and behavioural patterns [20]. For the latter, as sex differences in some adolescent health behaviors, such as physical activity, have been related to societal gender inequality, a society-level perspective should be adopted to address gender norms and disparities [24].

HBMI is a major risk factor for T2DM, and studies report that over 85% of children with T2DM are either overweight or obese at diagnosis [25]. The strong inverse association between BMI and age at diagnosis of T2DM also suggests an association between the duration of obesity and the risk of T2DM, which underscores the importance of preventing obesity in early childhood [26]. Adolescence has also been shown to be a highly dynamic period for weight gain, including incident obesity, and there is an association of weight gain during puberty and the risk of T2DM [13, 25]. The significance of these developmental periods reinforces the value of preventive interventions that take a life-course perspective to preventing overweight and obesity in young children as well as adolescents [26]. While most Western European countries have implemented obesity prevention policies, the strong relationship between the increasing rate of obesity in recent decades and the increasing incidence of T2DM among adolescents suggests much greater effort is indicated to slow the rising burden of DM across Europe [25]. In this context, DM among adolescents should be included within a social and commercial determinants of health framework, which will require a more comprehensive agenda aimed at the individual and societal level, as well as organizational and policy level. Greater emphasis may be warranted to ensure that preventive strategies focus on adolescents from lower socio-economic status (SES) and ethnic minorities as these are disproportionately affected by both T2DM and obesity [11] which are likely to have been exacerbated by the socio-economic, education and health inequalities caused by the COVID-19 pandemic [27].

These results show that while most countries have established DM strategies and national plans, a major barrier in the implementation of health policies is the absence of national registries. This averts the prioritisation of interventions which could be achieved through better understanding of trends in both forms of DM, and their associated complications. The lack of national registries is not restricted to T1DM, but it is this form of DM where the relevance of registries is most apparent, given the historic burden of complications that includes disabling sequelae and premature death, and the relevance of quality health services to reducing complications. The variability of estimates produced by different sources makes the absence of national registries immediately apparent. Although the global number of people with DM reported by GBD 2019 (463 million in 2019), the IDF 9th Atlas (460 million in 2019) and the NCD-Risk Collaboration (422 million in 2014) are aligned [15], greater variability for T1DM is reported [28]. GBD 2019 reported a prevalence of 1.4 million and 2.7 million cases of T1DM for 0–14 year-olds and 0–19 year-olds, while the IDF reported less than half the number, at 651,700 and 1.2 million respectively [28]. While this degree of variability will reflect differences in methods, case inclusion criteria and data sources used, it primarily reflects problems in primary data availability and quality; preliminary findings from the WHO Regional Office for Europe show that only two of the 24 Western European countries (Denmark and Sweden) included in our study have national diabetes registries. Ten countries have no registries, nine have registries for certain age groups or types of diabetes, and the situation was unknown in a further three countries [29]. Beyond disease surveillance, diabetes registries can be used to monitor aspects of clinical management, healthcare quality and healthcare costs, which are critical to many different aspects of governance and accountability, including policy implementation. Within and across countries, data harmonisation and standardisation should be prioritised as a strategy to enhance quality healthcare and patient outcomes, as well as a means of providing more precise knowledge of the direct and indirect economic burden of DM to the health system and society, which are estimated at about USD 189 billion in 2021 in the EUR Region [4].

This study shares the limitations of GBD 2019, which have been described in detail elsewhere [16, 19] Our analysis has several limitations related to the variation in the availability and quality of primary data for adolescents in general, including the paucity of data for 10–14 year-olds, and the particular paucity of DM data for 15–19 year-olds, thus there should be caution in interpretation [30–32]. The estimates of disease burden are surrounded by considerable uncertainties. Indeed, not all sources of uncertainty could be routinely captured in either the epidemiological or cause-of-death modelling processes. Differences in data availability among countries can generate difficulties in the interpretation of comparisons. Moreover, the wide uncertainty intervals for T2DM indicate caution in interpreting comparisons across countries. In this context, the addition of new and better-quality data will be essential to generate more reliable estimates in the future. Another limit is the unavailability of estimates for T2DM in adolescents <15 years old, as GBD uses the assumption that T2DM is >15 years old. This should be imputed to limited population-based data available for adolescents <15 years old. Additionally, considering that the PAF for risk factor-disease pairs are exclusively calculated for ages where both the risk and the disease are estimated, only high fasting plasma glucose and HBMI have been retrieved as risk factors for DM in the study population. However, as the proportion of T1DM and T2DM burden that is attributable to high fasting plasma glucose is always 100%, this metabolic risk factor has not been reported. HBMI was also only able to be analysed for the population aged 20–24 years old, due to the age restrictions reported above, notwithstanding that in terms of exposure values, all age groups <24 years showed an increase in HBMI in the 30 year period, finally leading to an increase in T2DM later in life (Supplementary Figures S7, S8). Other potentially important limitations relate to the lack of data on ethnicity and socio-economic status which are other risk factors for DM. Despite these limitations, a strength of this study is that it provides a comprehensive picture of the burden of DM across 24 Western European countries which will hopefully provide a benchmark for policymakers in regard to where investments are best prioritized.

In conclusion, this study describes striking increases in the incidence, prevalence, and disability burden of T1DM and T2DM among adolescents in Western Europe over the past 30 years. T1DM requires quality health system responses, which as reported in a recent scoping review [33] should expand implementation strategies, financial arrangements, and patient‐reported outcomes. For T2DM, policy is urgently needed on primary prevention as well as health system responses [34]. These should be based on understanding the relative impacts of the social and commercial determinants of health across the life course and extend beyond health service responses. Both forms of DM need further research to better understand their pathophysiology among adolescents and the interaction of genetics, puberty, and the environment. A specific focus on vulnerable populations appears indicated given the anticipated effect of intersectionalities (e.g., gender, ethnicity, low SES) on health outcomes, including mortality, as better appreciated across the COVID-19 pandemic.

## Group Members of GBD 2019 Europe Adolescent Diabetes

Hassan Abolhassani, Tigist Demssew Adane, Ayman Ahmed, Tareq Mohammed Ali AL-Ahdal, Catalina Liliana Andrei, Anton A Artamonov, Ashokan Arumugam, Muhammad Asaduzzaman, Prof Maciej Banach, Martina Barchitta, Prof Till Winfried Bärnighausen, Francesco Barone-Adesi, Luis Belo, Boris Bikbov, Aime Bonny, Prof Nikolay Ivanovich Briko, Prof Daniela Calina, Periklis Charalampous, Simiao Chen, Prof Rajiv Chowdhury, Prof Natália Cruz-Martins, Omid Dadras, Mostafa Dianatinasab, Monica Dinu, Arkadiusz Marian Dziedzic, Florian Fischer, Peter Andras Gaal, Bishal Gyawali, Ahmad Hammoud, Johannes Haubold, Prof Claudiu Herteliu, Salman Hussain, Irena M. Ilic, Prof Milena D. Ilic, Prof Gaetano Isola, Louis Jacob, Prof Mihajlo Jakovljevic, Elham Jamshidi, Haitham Khatatbeh, Prof Adnan Kisa, Prof Oleksii Korzh, Ai Koyanagi, Prof Carlo La Vecchia, Prof Tea Lallukka, Prof Anders O Larsson, Jacopo Lenzi, László Lorenzovici, Prof Stefan Lorkowski, Konstantinos Christos Makris, Hamid Reza Marateb, Andrea Maugeri, Tomislav Mestrovic, Junmei Miao Jonasson, Prof Tomasz Miazgowski, Irmina Maria Michalek, Kebede Haile Misgina, Prof Ulrich Otto Mueller, Francesk Mulita, Prof Christopher J L Murray, Prof Gabriele Nagel, Javaid Nauman, Ruxandra Irina Negoi, Prof Bogdan Oancea, Michal Ordak, Alicia Padron-Monedero, Tamás Palicz, Adrian Pana, Norberto Perico, Ionela-Roxana Petcu, Vera Pinheiro, Prof Maarten J Postma, Prof Giuseppe Remuzzi, Mónica Rodrigues, Prof Milena M. Santric-Milicevic, Art Schuermans, Rahman Shiri, Kerem Shuval, Prof Luís Manuel Lopes Rodrigues Silva, Prof Johan Sundström, Prof Rafael Tabarés-Seisdedos, Seyyed Mohammad Tabatabaei, Prof Tommi Juhani Vasankari, Ge+-3orgios-Ioannis Verras, Isidora S Vujcic, Rade Vukovic, Andrea Werdecker, Ronny Westerman, Prof Mikhail Sergeevich Zastrozhin, Magdalena Zielińska. Please see Supplementary Datasheet S2 for more detailed information about individual author contributions to the research, divided into the following categories: providing data or critical feedback on data sources; developing methods or computational machinery; providing critical feedback on methods or results; drafting the manuscript or revising it critically for important intellectual content; and managing the estimation or publications process. Please see Supplementary Datasheet S3 for the disclosures of the members of GBD 2019 Europe adolescent diabetes.
