# Supplementary material for: The Burden of Type 1 and Type 2 Diabetes Among Adolescents and Young Adults in 24 Western European Countries, 1990–2019: Results From the Global Burden of Disease Study 2019
Source: Int J Public Health. 2024 Feb 14;68:1606491. doi: 10.3389/ijph.2023.1606491 (PMC10899430; doi:10.3389/ijph.2023.1606491)
Supplement: Supplementary file 1 [file DataSheet4.docx]

## GBD 2019 Europe Adolescent Diabetes Collaborators

Hassan Abolhassani,^1,2^ Tigist Demssew Adane,^3^ Ayman Ahmed,^4,5^ Tareq Mohammed Ali AL-Ahdal,^6^ Catalina Liliana Andrei,^7^ Anton A Artamonov,^8^ Ashokan Arumugam,^9,10^ Muhammad Asaduzzaman,^11^ Prof Maciej Banach,^12,13^ Martina Barchitta,^14^ Prof Till Winfried Bärnighausen,^6,15^ Francesco Barone-Adesi,^16^ Luis Belo,^17,18^ Boris Bikbov,^19^ Aime Bonny,^20,21^ Prof Nikolay Ivanovich Briko,^22^ Prof Daniela Calina,^23^ Periklis Charalampous,^24^ Simiao Chen,^6^ Prof Rajiv Chowdhury,^25,26^ Prof Natália Cruz-Martins,^27,28^ Omid Dadras,^29,30^ Mostafa Dianatinasab,^31,32^ Monica Dinu,^33^ Arkadiusz Marian Dziedzic,^34^ Florian Fischer,^35^ Peter Andras Gaal,^36,37^ Bishal Gyawali,^38^ Ahmad Hammoud,^39^ Johannes Haubold,^40,41^ Prof Claudiu Herteliu,^42,43^ Salman Hussain,^44,45^ Irena M. Ilic,^46^ Prof Milena D. Ilic,^47^ Prof Gaetano Isola,^48^ Louis Jacob,^49,50^ Prof Mihajlo Jakovljevic,^51,52^ Elham Jamshidi,^53^ Haitham Khatatbeh,^54^ Prof Adnan Kisa,^55,56^ Prof Oleksii Korzh,^57^ Ai Koyanagi,^58^ Prof Carlo La Vecchia,^59^ Prof Tea Lallukka,^60^ Prof Anders O Larsson,^61,62^ Jacopo Lenzi,^63^ László Lorenzovici,^64,65^ Prof Stefan Lorkowski,^66,67^ Konstantinos Christos Makris,^68^ Hamid Reza Marateb,^69,70^ Andrea Maugeri,^71^ Tomislav Mestrovic,^72,73^ Junmei Miao Jonasson,^74^ Prof Tomasz Miazgowski,^75^ Irmina Maria Michalek,^76,77^ Kebede Haile Misgina,^78,79^ Prof Ulrich Otto Mueller,^80,81^ Francesk Mulita,^82,83^ Prof Christopher J L Murray,^73,84^ Prof Gabriele Nagel,^85^ Javaid Nauman,^86,87^ Ruxandra Irina Negoi,^88,89^ Prof Bogdan Oancea,^90^ Michal Ordak,^91^ Alicia Padron-Monedero,^92^ Tamás Palicz,^36,93^ Adrian Pana,^94,95^ Norberto Perico,^96^ Ionela-Roxana Petcu,^42^ Vera Pinheiro,^97,98^ Prof Maarten J Postma,^99,100^ Prof Giuseppe Remuzzi,^96^ Mónica Rodrigues,^101^ Prof Milena M. Santric-Milicevic,^46,102^ Art Schuermans,^103,104^ Rahman Shiri,^105^ Kerem Shuval,^106^ Prof Luís Manuel Lopes Rodrigues Silva,^107,108^ Prof Johan Sundström,^61,109^ Prof Rafael Tabarés-Seisdedos,^110,111^ Seyyed Mohammad Tabatabaei,^112,113^ Prof Tommi Juhani Vasankari,^114,115^ Georgios-Ioannis Verras,^82,116^ Isidora S Vujcic,^46^ Rade Vukovic,^117,118^ Andrea Werdecker,^119^ Ronny Westerman,^120^ Prof Mikhail Sergeevich Zastrozhin,^121,122^ Magdalena Zielińska.^123^

## Affiliations

^1^Research Center for Immunodeficiencies, Tehran University of Medical Sciences, Tehran, Iran; ^2^Department of Medical Biochemistry and Biophysics, Karolinska Institute, Stockholm, Sweden; ^3^Department of Clinical and Psychosocial Epidemiology, University of Groningen, Groningen, Netherlands; ^4^Institute of Endemic Diseases, University of Khartoum, Khartoum, Sudan; ^5^Swiss Tropical and Public Health Institute, University of Basel, Basel, Switzerland; ^6^Heidelberg Institute of Global Health (HIGH), Heidelberg University, Heidelberg, Germany; ^7^Cardiology Department, Carol Davila University of Medicine and Pharmacy, Bucharest, Romania; ^8^Department of Biophysics, Russian Academy of Sciences, Moscow, Russia; ^9^Department of Physiotherapy, University of Sharjah, Sharjah, United Arab Emirates; ^10^Department of Community Medicine and Rehabilitation, Umeå University, Umea, Sweden; ^11^Department of Community Medicine and Global Health, University of Oslo, Oslo, Norway; ^12^Department of Hypertension, Medical University of Lodz, Lodz, Poland; ^13^Polish Mothers' Memorial Hospital Research Institute, Lodz, Poland; ^14^Department of Medical and Surgical Sciences and Advanced Technologies "GF Ingrassia", University of Catania, Catania, Italy; ^15^T.H. Chan School of Public Health, Harvard University, Boston, MA, USA; ^16^Department of Translational Medicine, University of Eastern Piedmont, Novara, Italy; ^17^Biological Sciences Department, University of Porto, Porto, Portugal; ^18^Research Unit on Applied Molecular Biosciences (UCIBIO), University of Porto, Porto, Portugal; ^19^Scientific-Tools, Bergamo, Italy; ^20^Faculty of Medicine and Pharmaceutical Sciences, University of Douala, Douala, Cameroon; ^21^Department of Cardiology, Centre Hospitalier Montfermeil (Montfermeil Hospital Center), Montfermeil, France; ^22^Department of Epidemiology and Evidence-Based Medicine, I.M. Sechenov First Moscow State Medical University, Moscow, Russia; ^23^Department of Clinical Pharmacy, University of Medicine and Pharmacy of Craiova, Craiova, Romania; ^24^Department of Public Health, Erasmus University Medical Center, Rotterdam, Netherlands; ^25^Florida International University, Florida International University, Miami, FL, USA; ^26^Department of Epidemiology, University of Bern, Bern, Switzerland; ^27^Department of Therapeutic and Diagnostic Technologies, Cooperativa de Ensino Superior Politécnico e Universitário (Polytechnic and University Higher Education Cooperative), Gandra, Portugal; ^28^Institute for Research and Innovation in Health, University of Porto, Porto, Portugal; ^29^Department of Addiction Medicine, Haukland University Hospital, Bergen, Norway; ^30^Department of Global Public Health and Primary Care, University of Bergen, Bergen, Norway; ^31^Department of Epidemiology, Sunway University, Malaysia; ^32^Department of Epidemiology, Maastricht University, Netherlands; ^33^Department of Experimental and Clinical Medicine, University of Florence, Florence, Italy; ^34^Department of Conservative Dentistry with Endodontics, Medical University of Silesia, Katowice, Poland; ^35^Institute of Public Health, Charité Universitätsmedizin Berlin (Charité Medical University Berlin), Berlin, Germany; ^36^Health Services Management Training Centre, Semmelweis University, Budapest, Hungary; ^37^Department of Applied Social Sciences, Sapientia Hungarian University of Transylvania, Târgu-Mureş, Romania; ^38^Department of Public Health, University of Copenhagen, Copenhagen, Denmark; ^39^Department of Medical and Technical Information Technology, Bauman Moscow State Technical University, Moscow, Russia; ^40^Department of Diagnostic and Interventional Radiology and Neuroradiology, University Hospital Essen, Essen, Germany; ^41^Institute of Artificial Intelligence in Medicine, University Hospital Essen, Essen, Germany; ^42^Department of Statistics and Econometrics, Bucharest University of Economic Studies, Bucharest, Romania; ^43^School of Business, London South Bank University, London, UK; ^44^Czech National Centre for Evidence-Based Healthcare and Knowledge Translation, Masaryk University, Brno, Czech Republic; ^45^Institute of Biostatistics and Analyses, Masaryk University, Brno, Czech Republic; ^46^Faculty of Medicine, University of Belgrade, Belgrade, Serbia; ^47^Department of Epidemiology, University of Kragujevac, Kragujevac, Serbia; ^48^Department of General Surgery and Surgical-Medical Specialties, University of Catania, Catania, Italy; ^49^Research and Development Unit, Biomedical Research Networking Center for Mental Health Network (CiberSAM), Sant Boi de Llobregat, Spain; ^50^Faculty of Medicine, University of Versailles Saint-Quentin-en-Yvelines, Montigny-le-Bretonneux, France; ^51^Medical and Health Sciences Section, The World Academy of Sciences UNESCO, Trieste, Italy; ^52^Shaanxi University of Technology, Hanzhong, China; ^53^Johns Hopkins University, Baltimore, MD, USA; ^54^Faculty of Nursing, Jerash University, Jerash, Jordan; ^55^School of Health Sciences, Kristiania University College, Oslo, Norway; ^56^Department of International Health and Sustainable Development, Tulane University, New Orleans, LA, USA; ^57^Department of General Practice and Family Medicine, Kharkiv National Medical University, Kharkiv, Ukraine; ^58^San Juan de Dios Sanitary Park, Barcelona, Spain; ^59^Department of Clinical Sciences and Community Health, University of Milan, Milan, Italy; ^60^Department of Public Health, University of Helsinki, Helsinki, Finland; ^61^Department of Medical Sciences, Uppsala University, Uppsala, Sweden; ^62^Department of Clinical Chemistry and Pharmacology, Uppsala University Hospital, Uppsala, Sweden; ^63^Department of Biomedical and Neuromotor Sciences, University of Bologna, Bologna, Italy; ^64^Department of Health Economics, Syreon Research Romania, Targu Mures, Romania; ^65^Department of Doctoral Studies, George Emil Palade University of Medicine, Pharmacy, Science, and Technology of Targu Mures, Targu Mures, Romania; ^66^Institute of Nutritional Sciences, Friedrich Schiller University Jena, Jena, Germany; ^67^Competence Cluster for Nutrition and Cardiovascular Health (nutriCARD), Jena, Germany; ^68^Cyprus International Institute for Environmental and Public Health, Cyprus University of Technology, Limassol, Cyprus; ^69^Biomedical Engineering Research Center (CREB), Universitat Politècnica de Catalunya, Barcelona, Spain; ^70^Biomedical Engineering, University of Isfahan, Isfahan, Iran; ^71^Department GF Ingrassia, University of Catania, Catania, Italy; ^72^University Centre Varazdin, University North, Varazdin, Croatia; ^73^Institute for Health Metrics and Evaluation, University of Washington, Seattle, WA, USA; ^74^School of Public Health and Community Medicine, University of Gothenburg, Gothenburg, Sweden; ^75^Department of Propedeutics of Internal Diseases & Arterial Hypertension, Pomeranian Medical University, Szczecin, Poland; ^76^Department of Epidemiology, Maria Sklodowska-Curie National Research Institute of Oncology, Warsaw, Poland; ^77^National Cancer Registry, Maria Sklodowska-Curie National Research Institute of Oncology, Warsaw, Poland; ^78^Department of Epidemiology, University of Groningen, Groningen, Netherlands; ^79^Department of Public Health, Aksum University, Axum, Ethiopia; ^80^Federal Institute for Population Research, Wiesbaden, Germany; ^81^Center for Population and Health, Wiesbaden, Germany; ^82^Department of Surgery, General University Hospital of Patras, Patras, Greece; ^83^Faculty of Medicine, University of Thessaly, Larissa, Greece; ^84^Department of Health Metrics Sciences, School of Medicine, University of Washington, Seattle, WA, USA; ^85^Institute of Epidemiology and Medical Biometry, Ulm University, Ulm, Germany; ^86^College of Medicine and Health Sciences, United Arab Emirates University, Al Ain, United Arab Emirates; ^87^Department of Circulation and Medical Imaging, Norwegian University of Science and Technology, Trondheim, Norway; ^88^Department of Anatomy and Embryology, Carol Davila University of Medicine and Pharmacy, Bucharest, Romania; ^89^Department of Cardiology, Cardio-Aid, Bucharest, Romania; ^90^Department of Applied Economics and Quantitative Analysis, University of Bucharest, Bucharest, Romania; ^91^Department of Pharmacotherapy and Pharmaceutical Care, Medical University of Warsaw, Warsaw, Poland; ^92^National School of Public Health, Institute of Health Carlos III, Madrid, Spain; ^93^Hungarian Health Management Association, Hungarian Health Management Association, Budapest, Hungary; ^94^Department of Public Health, Babes Bolyai University, Cluj Napoca, Romania; ^95^Department of Health Metrics, Center for Health Outcomes & Evaluation, Bucharest, Romania; ^96^Mario Negri Institute for Pharmacological Research, Bergamo, Italy; ^97^Public Health Unit, Local Health Unit of Matosinhos, Matosinhos, Portugal; ^98^Faculty of Medicine, University of Porto, Porto, Portugal; ^99^University Medical Center Groningen, University of Groningen, Groningen, Netherlands; ^100^Center of Excellence in Higher Education for Pharmaceutical Care Innovation, Universitas Padjadjaran (Padjadjaran University), Bandung, Indonesia; ^101^Department of Geography and Demography, University of Coimbra, Coimbra, Portugal; ^102^School of Public Health and Health Management, University of Belgrade, Belgrade, Serbia; ^103^Cardiovascular Research Center, Massachusetts General Hospital, Cambridge, MA, USA; ^104^Department of Cardiovascular Sciences, Katholieke Universiteit Leuven, Leuven, Belgium; ^105^Finnish Institute of Occupational Health, Helsinki, Finland; ^106^The Cooper Institute, Dallas, TX, USA; ^107^Center of Potential and Innovation of Natural Resources, Polytechnic Institute of Guarda, Guarda, Portugal; ^108^Health Sciences Research Centre, University of Beira Interior, Covilhã, Portugal; ^109^The George Institute for Global Health, Sydney, NSW, Australia; ^110^Department of Medicine, University of Valencia, Valencia, Spain; ^111^Carlos III Health Institute, Biomedical Research Networking Center for Mental Health Network (CiberSAM), Madrid, Spain; ^112^Department of Medical Informatics, Mashhad University of Medical Sciences, Mashhad, Iran; ^113^Clinial Research Development Unit, Mashhad University of Medical Sciences, Mashhad, Iran; ^114^UKK Institute, Tampere, Finland; ^115^Faculty of Medicine and Health Technology, Tampere University, Tampere, Finland; ^116^College of Medicine and Veterinary Medicine, University of Edinburgh, Edinburgh, UK; ^117^School of Medicine, University of Belgrade, Belgrade, Serbia; ^118^Department of Pediatric Endocrinology, Mother and Child Healthcare Institute of Serbia "Dr Vukan Cupic", Belgrade, Serbia; ^119^Demographic Change and Aging Research Area, Federal Institute for Population Research, Wiesbaden, Germany; ^120^Competence Center of Mortality-Follow-Up of the German National Cohort, Federal Institute for Population Research, Wiesbaden, Germany; ^121^Department of Bioengineering and Therapeutic Sciences, University of California San Francisco, San Francisco, CA, USA; ^122^Addictology Department, Russian Medical Academy of Continuous Professional Education, Moscow, Russia; ^123^Department of Biochemistry and Pharmacogenomics, Medical University of Warsaw, Warsaw, Poland.
