# Supplementary material for: The Burden of Type 1 and Type 2 Diabetes Among Adolescents and Young Adults in 24 Western European Countries, 1990–2019: Results From the Global Burden of Disease Study 2019
Source: Int J Public Health. 2024 Feb 14;68:1606491. doi: 10.3389/ijph.2023.1606491 (PMC10899430; doi:10.3389/ijph.2023.1606491)
Supplement: Supplementary file 2 [file DataSheet3.docx]

**Disclosures**

T W Bärnighausen reports other financial or non-financial support from Fondation Botnar and the Harvard T H Chan School of Public Health; grants from the National Institutes of Health (NIH), Wellcome Trust, German National Science Foundation, German Ministry of Education and Research, Volkswagen Foundation, European Union Horizon Europe, German Ministry of the Environment, Alexander von Humboldt Foundation, Else-Kröner-Fresenius-Foundation, and the International Vaccine Institute; stock or stock options with CHEERS; outside the submitted work. L Belo reports financial interests and support from FCT in the scope of the project UIDP/04378/2020 and UIDB/04378/2020 of UCIBIO and the project LA/P/0140/2020 of i4HB; outside the submitted work. B Bikbov reports grants or contracts from the European Commission; support for attending meetings and/or travel from the European Renal Association; Leadership or fiduciary role in an advocacy group, unpaid with the International Society of Nephrology; and other non-financial interests with Scientific-Tools.org; outside the submitted work. C Herteliu reports grants or contracts from the Romanian Ministry of Research Innovation and Digitalization, MCID, project number ID-585-CTR-42-PFE-2021, A grant of the European Commission Horizon 4P-CAN (Personalised Cancer Primary Prevention Research through Citizen Participation and Digitally Enabled Social Innovation), Project “Societal and Economic Resilience within multi-hazards environment in Romania” funded by European Union – NextgenerationEU and Romanian Government, under National Recovery and Resilience Plan for Romania, contract no.760050/ 23.05.2023, cod PNRR-C9-I8-CF 267/ 29.11.2022, through the Romanian Ministry of Research, Innovation and Digitalization, within Component 9, Investment I8, and Project “A better understanding of socio-economic systems using quantitative methods from Physics'' funded by European Union – NextgenerationEU and Romanian Government, under National Recovery and Resilience Plan for Romania, contract no.760034/ 23.05.2023, cod PNRR-C9-I8-CF 255/ 29.11.2022, through the Romanian Ministry of Research, Innovation and Digitalization, within Component 9, Investment I8; outside the submitted work. I Ilic reports other financial or non-financial support from the Ministry of Education, Science and Technological Development, Republic of Serbia (project No 175042, 2011-2023); outside the submitted work. M Ilic reports other financial or non-financial support from the Ministry of Education, Science and Technological Development, Republic of Serbia (project No 451-03-47/2023-01/200111); outside the submitted work. S Lorkowski reports grants or contracts from Akcea Therapeutics (Germany) and DSM Nutritional Products; consulting fees from Danone, Novartis Pharma, and Swedish Orphan Biovitrum (SOBI); Payment or honoraria for lectures, presentations, speakers bureaus, manuscript writing or educational events from Akcea Therapeutics Germany, AMARIN Germany, Amedes Holding, AMGEN, Berlin-Chemie, Boehringer Ingelheim Pharma, Daiichi Sankyo Deutschland, Danone, Hubert Burda Media Holding, Janssen-Cilag, Lilly Deutschland, Novartis Pharma, Novo Nordisk Pharma, Roche Pharma, Sanofi-Aventis, SYNLAB Holding Deutschland & SYNLAB Akademie; Support for attending meetings/travel from AMGEN; Participation on a Data Safety Monitoring Board or Advisory Board with Akcea Therapeutics Germany, AMGEN, Daiichi Sankyo Deutschland, Novartis Pharma, and Sanofi-Aventis; outside the submitted work. H R Marateb reports other financial or non-financial support from Universitat Politècnica de Catalunya-Barcelona Tech (UPC) (The Beatriu de Pinós post-doctoral programme from the Office of the Secretary of Universities and Research from the Ministry of Business and Knowledge of the Government of Catalonia programme: 2020 BP 00261). L Monasta reports support for the present manuscript from the Ministry of Health (Ricerca Corrente 34/2017) payments made to the Institute for Maternal and Child Health IRCCS Burlo Garofolo. U O Mueller reports grants or contracts from the German Federal Ministry for Education and Research paid to their institution; Payment or honoraria for lectures, presentations, speakers bureaus, manuscript writing or educational events from Poster Prize, Annual Meeting German Society for Internal Medicine; support for attending meetings and travel from their institution; outside the submitted work. L M L R Da Silva reports grants or contracts from project code CENTRO-04-3559-FSE-000162, Fundo Social Europeu (FSE); outside the submitted work. L K Stafford reports support for the present manuscript from the Bill and Melinda Gates Foundation and the Institute for Health Metrics and Evaluation. J Sundstrom reports stock or stock options as a shareholder Anagram Kommunikation AB and Symptoms Europe AB, outside the submitted work. R Tabarés-Seisdedos reports grants or contracts from Spanish Ministry of Science and Innovation, Institute of Health Carlos III (PID2021-129099OB-I00) and GENERALITAT VALENCIANA (CIPROM/2022/58) all paid to their institution (University of Valencia); outside the submitted work. M Zielińska reports other financial interests as an AstraZeneca employee; outside the submitted work.
