# Supplementary material for: The Burden of Type 1 and Type 2 Diabetes Among Adolescents and Young Adults in 24 Western European Countries, 1990–2019: Results From the Global Burden of Disease Study 2019
Source: Int J Public Health. 2024 Feb 14;68:1606491. doi: 10.3389/ijph.2023.1606491 (PMC10899430; doi:10.3389/ijph.2023.1606491)
Supplement: Supplementary file 3 [file DataSheet1.PDF]

The burden of type 1 and type 2 diabetes among adolescents in 24 Western European countries, 1990-2019: results from the Global Burden of Disease Study 2019

Supplementary file

Supplementary Figure 1. Prevalence rate per 100,000 population for DM among 10-24-year-olds, from 1990-2019, in 24 Western European countries........ 2

Supplementary Figure 2. Prevalence rate per 100,000 (with 95% UI) of T1DM and T2DM, in 24 Western European countries, in 2019, by sex and age 3

Supplementary Figure 3. Incidence rate per 100,000 of T1DM and T2DM, in 24 Western European countries, in 2019, by sex and age........ 5

Supplementary Figure 4. Incidence rate per 100,000 population (with 95% UI) of T1DM and T2DM, in 10-24-year-olds of both sexes, in 2019, by country 7

Supplementary Figure 5. YLDs rate per 100,000 population (with 95% UI), in 10-24-year-olds of both sexes, in 2019, by country........ 9

Supplementary Figure 6. YLDs rate per 100,000 population (with 95% UI) for T2DM attributed to HMBI, in 20-24-year-olds, in 2019, by country and sex........ 11

Supplementary Figure 7. Summary exposure value rate per 100,000 attributed to HBMI in Western Europe, in 2019, by age groups (<24 years) and sex........ 12

Supplementary Figure 8. Summary exposure value rate per 100,000 attributed to HBMI in Western Europe, by age groups (<24 years), sex and trend over time (1990-2019) 13

Supplementary Table 1. T1DM and T2DM prevalence, incidence and YLDs case numbers (with 95%UI), in 2019, in 24 Western European countries, by sex and age........ 14

Supplementary Table 2. T1DM and T2DM prevalence, incidence and YLDs rate per 100,000 population (with 95%UI) in 2019, in 24 Western European countries, by sex and age 15

Supplementary Table 3. T1DM and T2DM incidence rate per 100,000 population (with 95%UI), in Western Europe and by 24 countries, in 1990 and 2019, with percentage change by time (with 95%UI) 16

Supplementary Table 4. YLDs rate per 100,000 for T2DM (with 95%UI), attributed to HBMI, in Western Europe and by 24 countries, in 1990 and 2019, with percentage change by time (with 95%UI) 17

Supplementary Figure 1. Prevalence rate per 100,000 population for DM among 10-24-year-olds, from 1990-2019, in 24 Western European countries

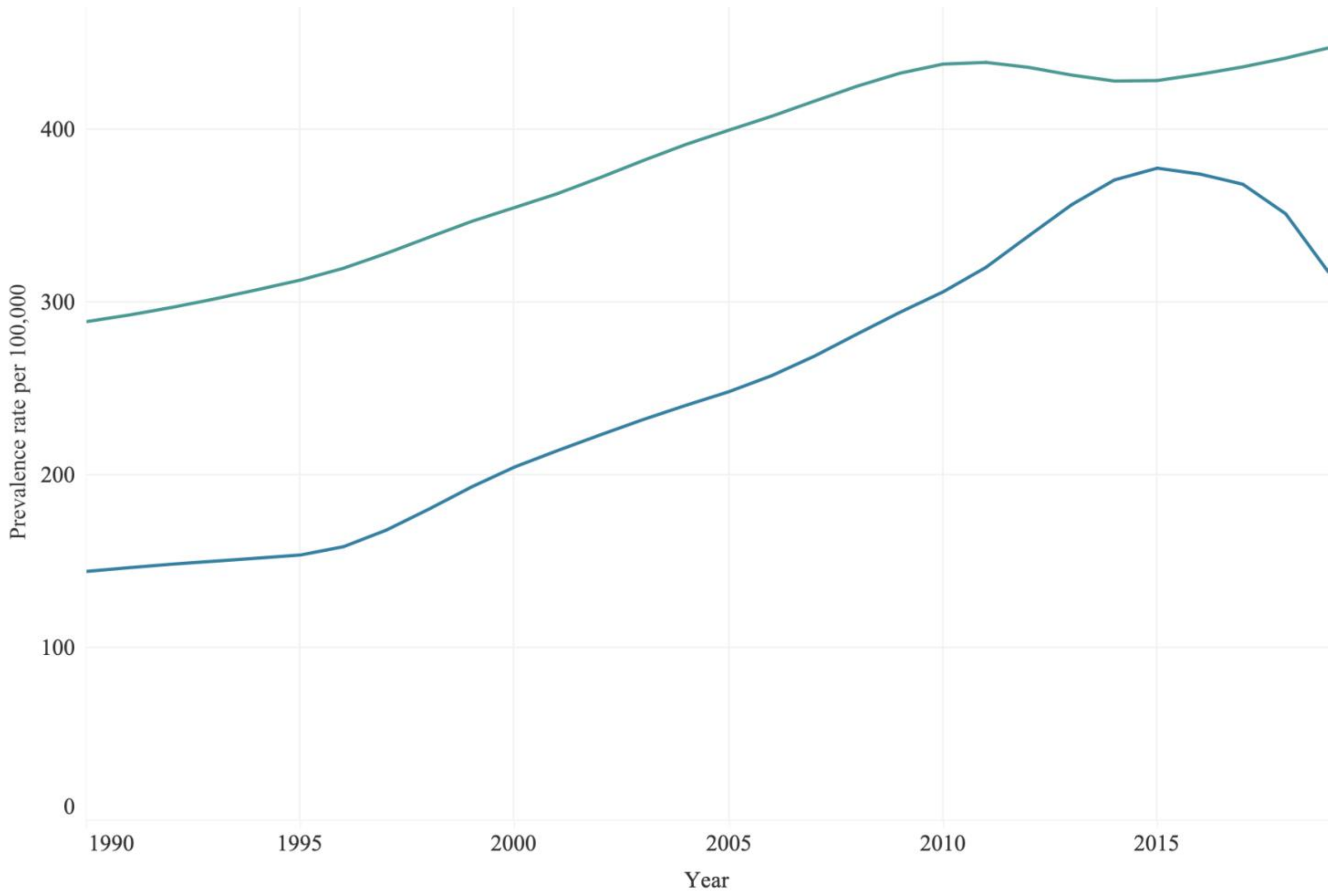

Abbreviation: DM=Diabetes mellitus; T1DM=Type 1 diabetes mellitus; T2DM=Type 2 diabetes mellitus

DM
T1DM
T2DM

Supplementary Figure 2. Prevalence rate per 100,000 (with 95% UI) of T1DM and T2DM, in 24 Western European countries, in 2019, by sex and age

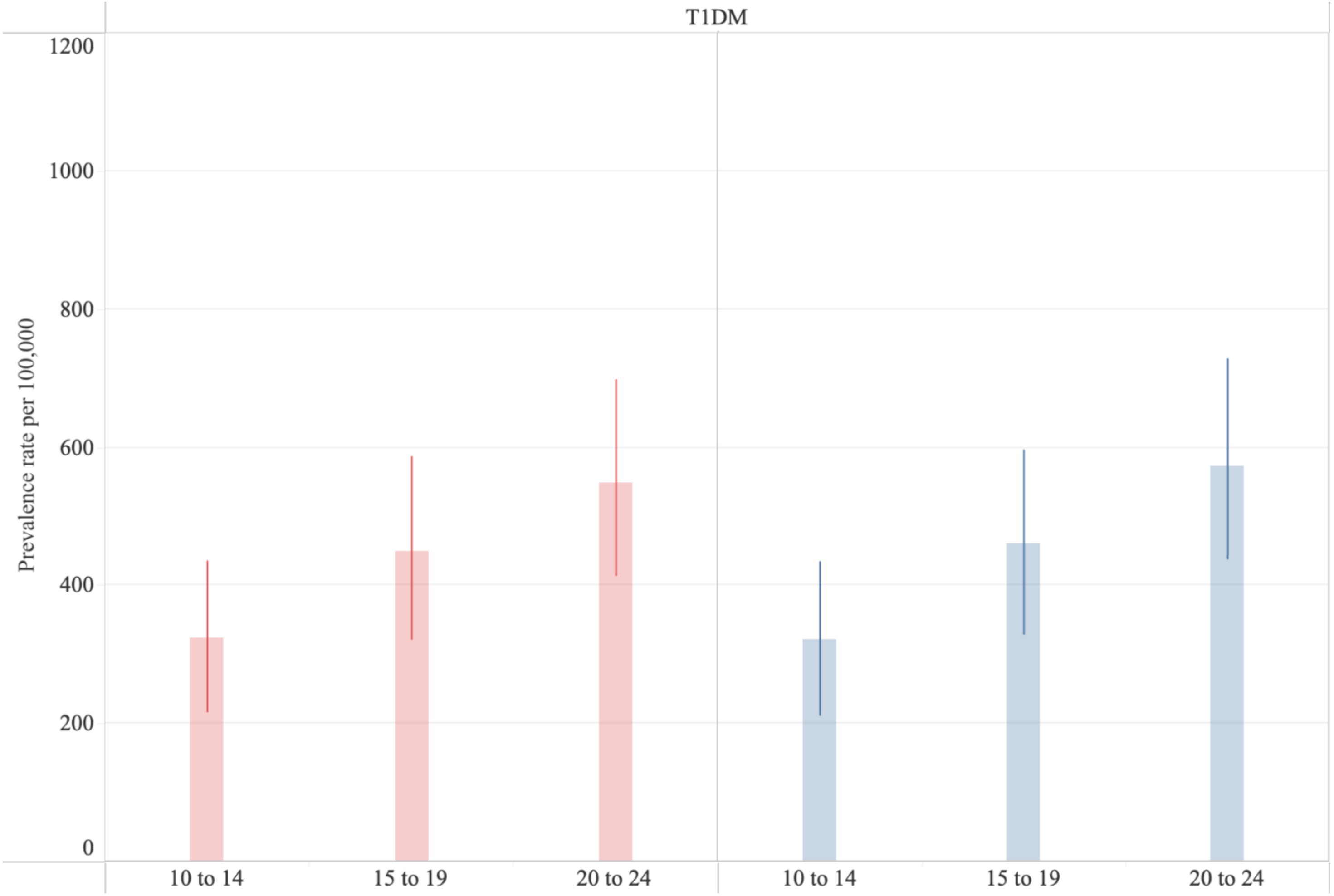

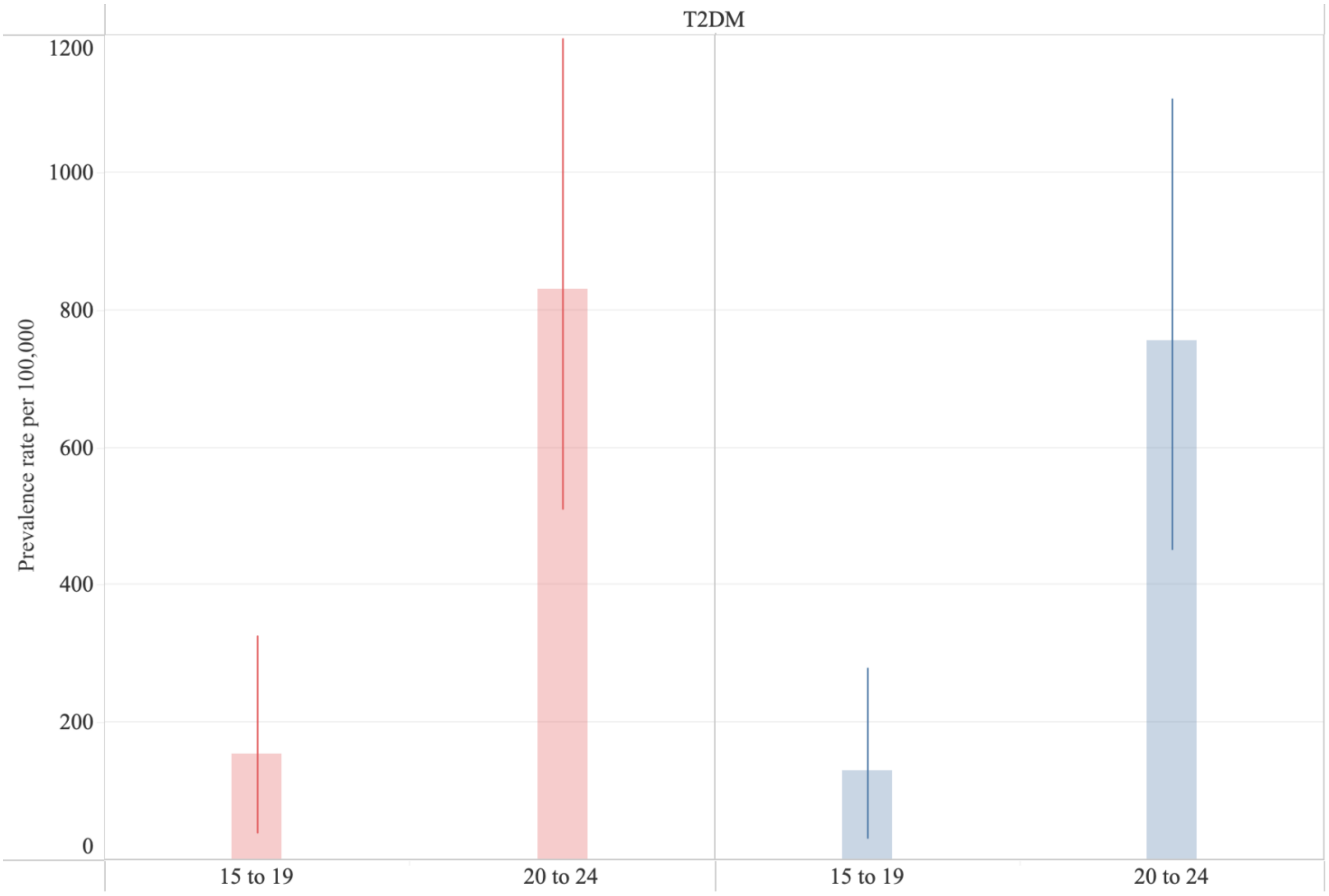

Abbreviation: T1DM=Type 1 diabetes mellitus; T2DM=Type 2 diabetes mellitus; 95% UI=95% uncertainty interval
*Note: GBD study 2019 assumes that under 15 years all DM is T1DM

Sex
Female
Male

Supplementary Figure 3. Incidence rate per 100,000 of T1DM and T2DM, in 24 Western European countries, in 2019, by sex and age

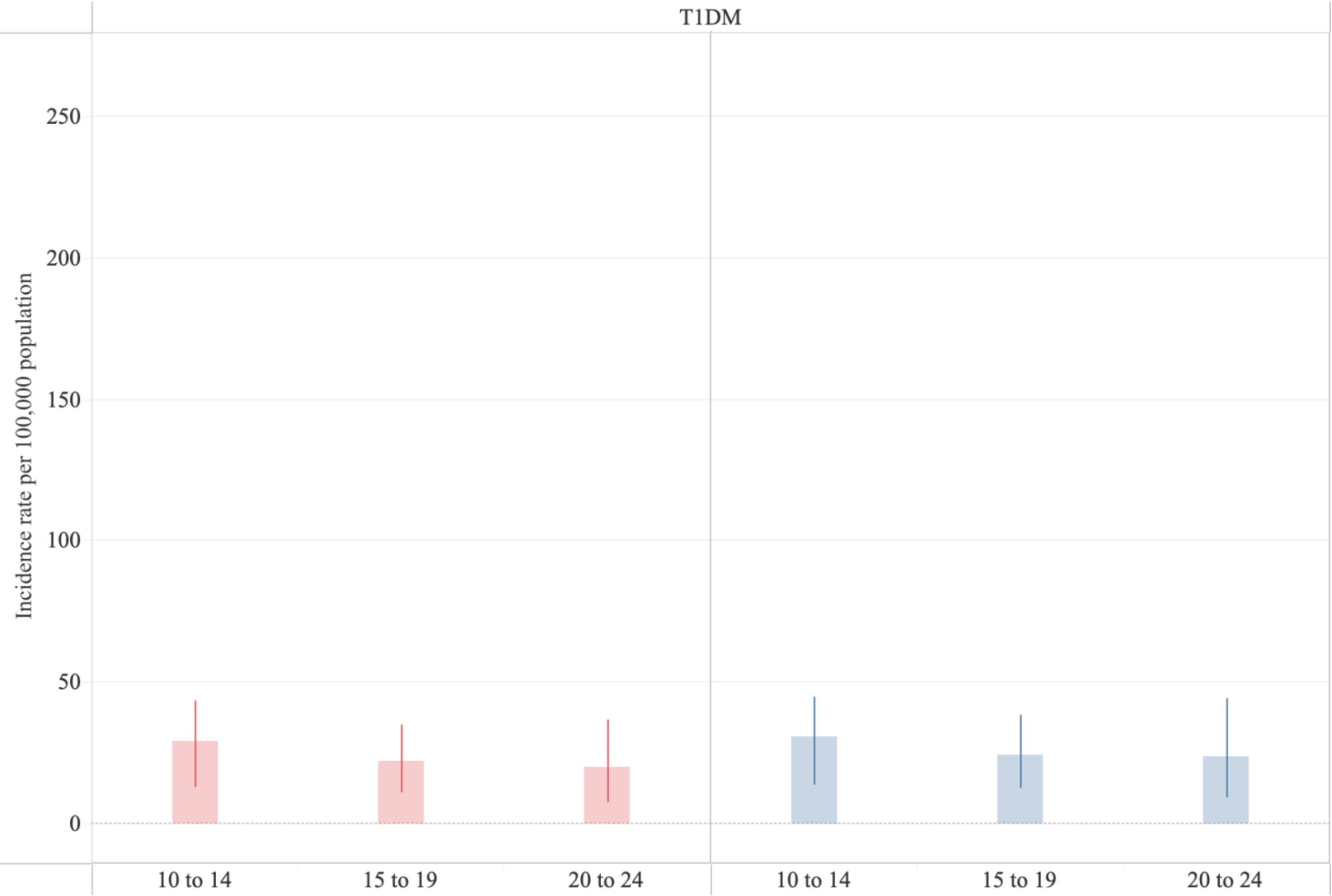

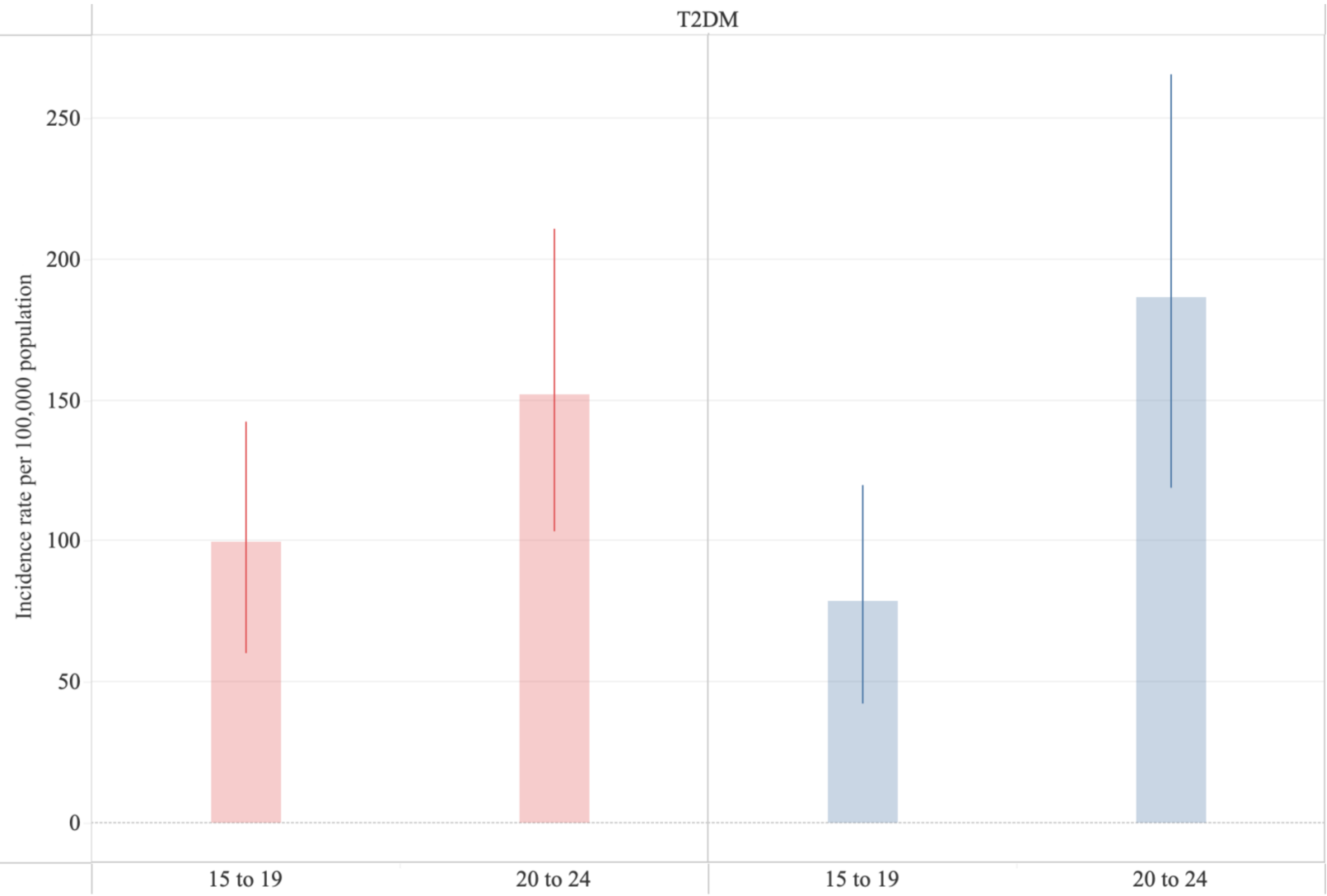

Abbreviations: T1DM=Type 1 diabetes mellitus; T2DM=Type 2 diabetes mellitus
*Note: GBD study 2019 assumes that under 15 years all DM is T1DM

Sex
Female
Male

Supplementary Figure 4. Incidence rate per 100,000 population (with 95% UI) of T1DM and T2DM, in 10-24-year-olds of both sexes, in 2019, by country

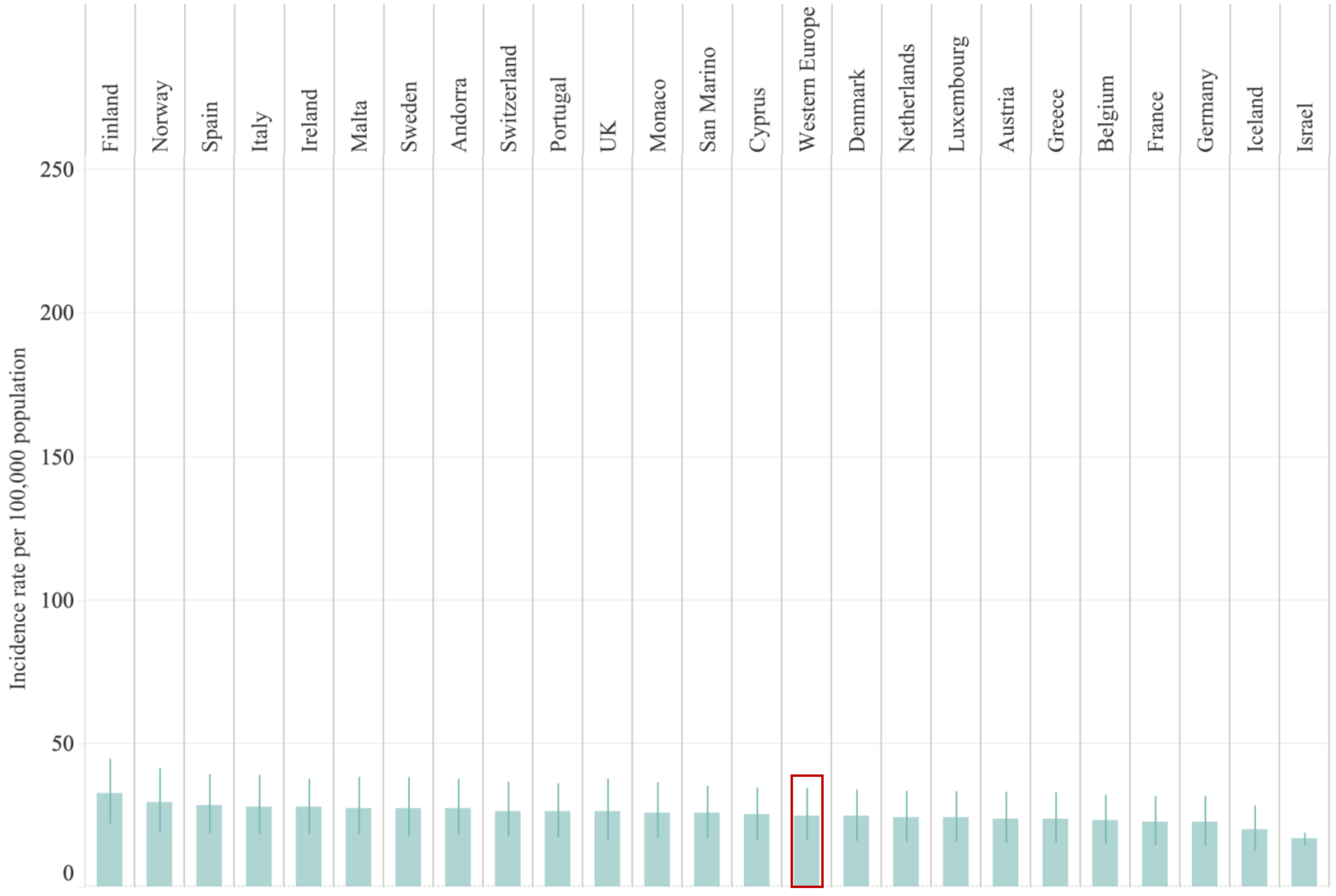

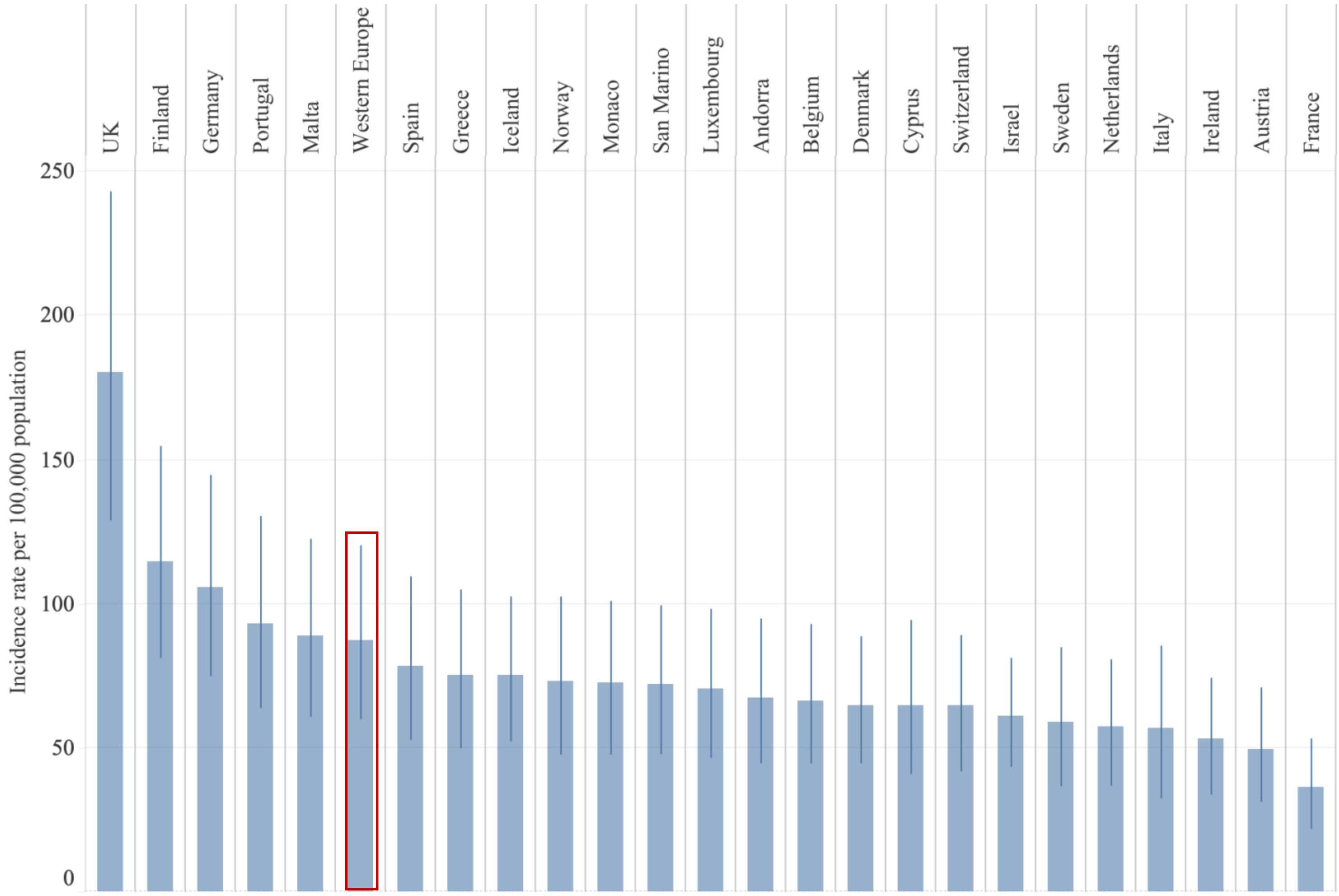

Abbreviation: T1DM=Type 1 diabetes mellitus; T2DM=Type 2 diabetes mellitus; 95% UI=95% uncertainty interval

DM
T1DM
T2DM

Supplementary Figure 5. YLDs rate per 100,000 population (with 95% UI), in 10-24-year-olds of both sexes, in 2019, by country

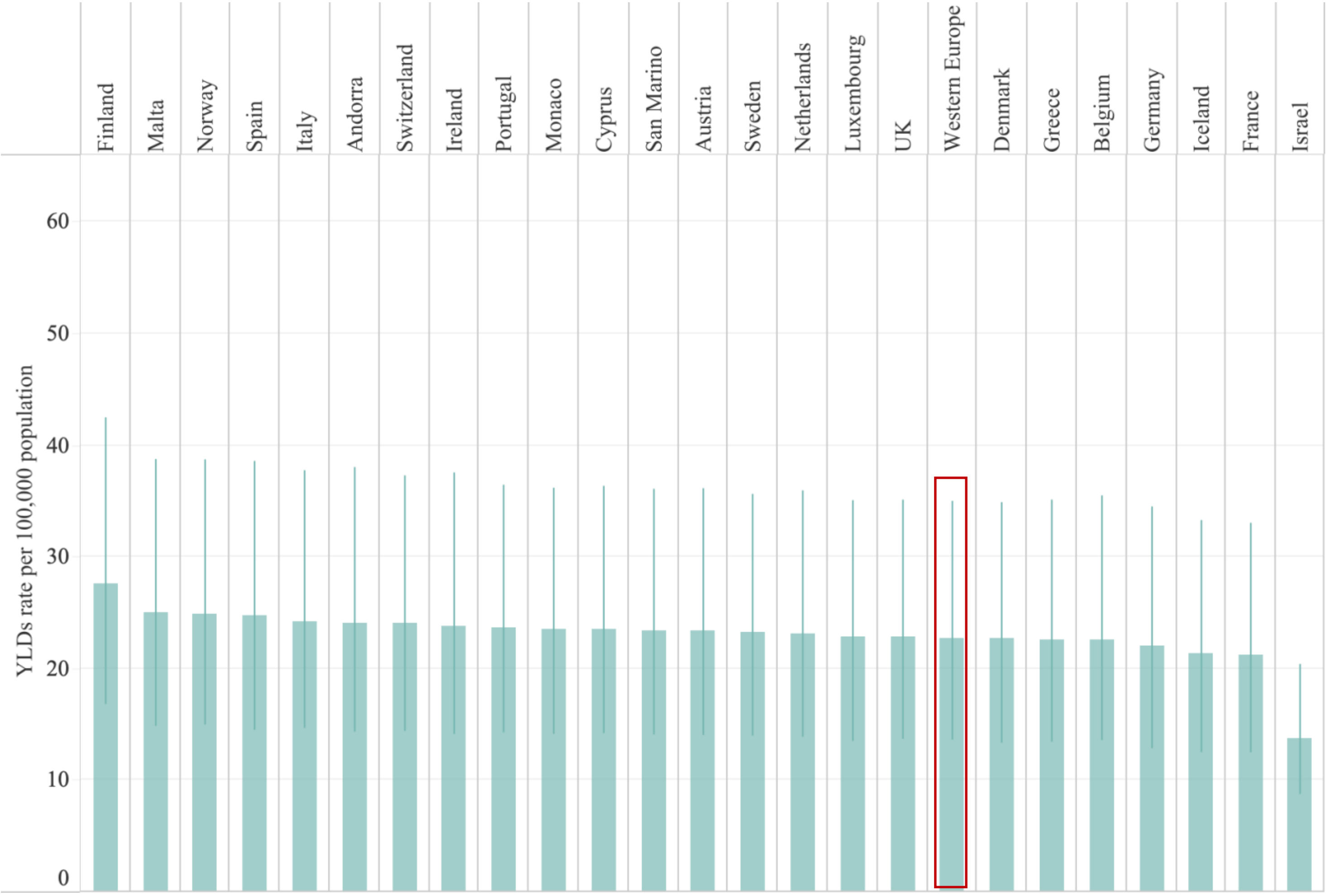

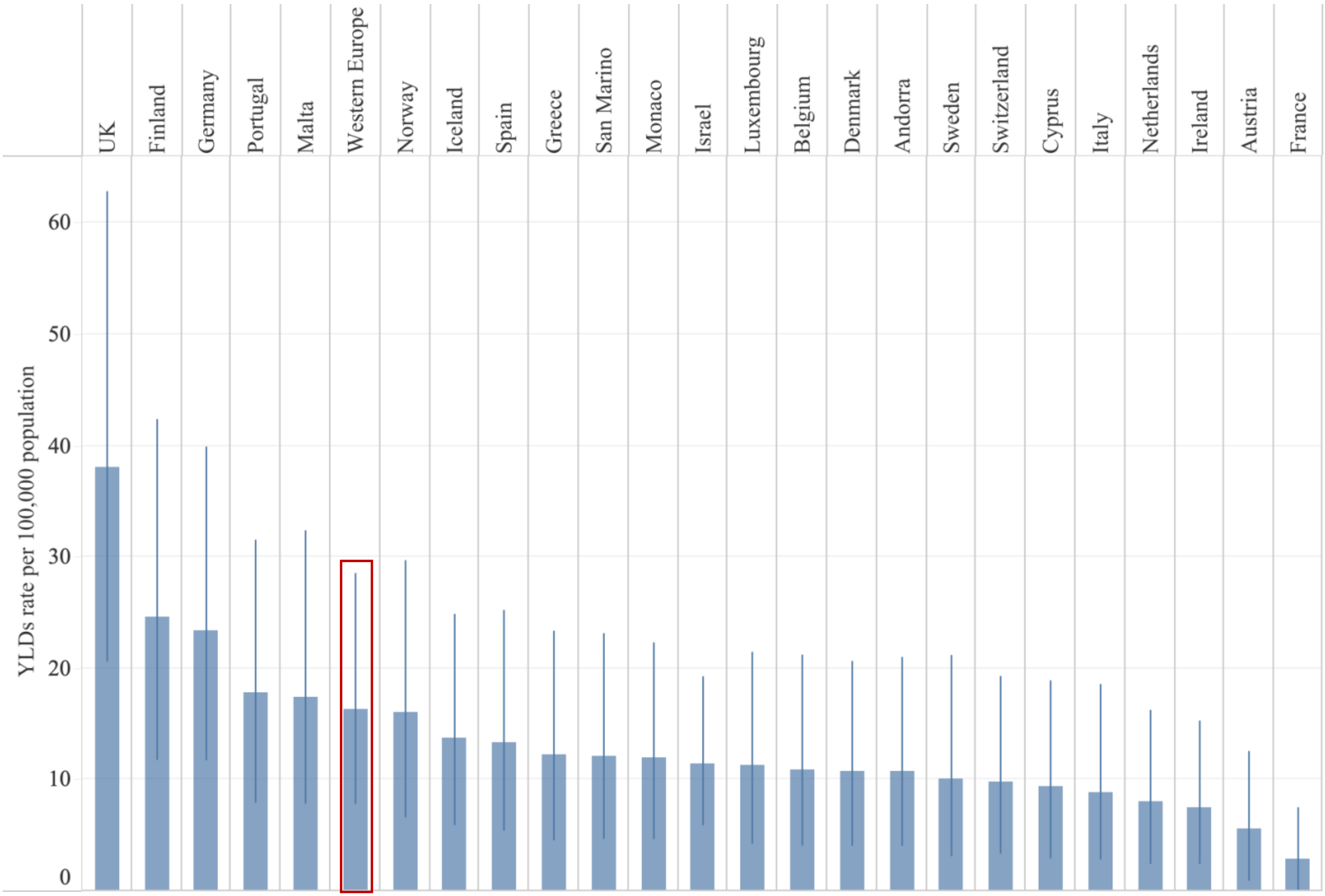

Abbreviation: T1DM=Type 1 diabetes mellitus; T2DM=Type 2 diabetes mellitus; 95% UI=95% uncertainty interval

DM
T1DM
T2DM

Supplementary Figure 6. YLDs rate per 100,000 population (with 95% UI) for T2DM attributed to HMBI, in 20-24-year-olds, in 2019, by country and sex

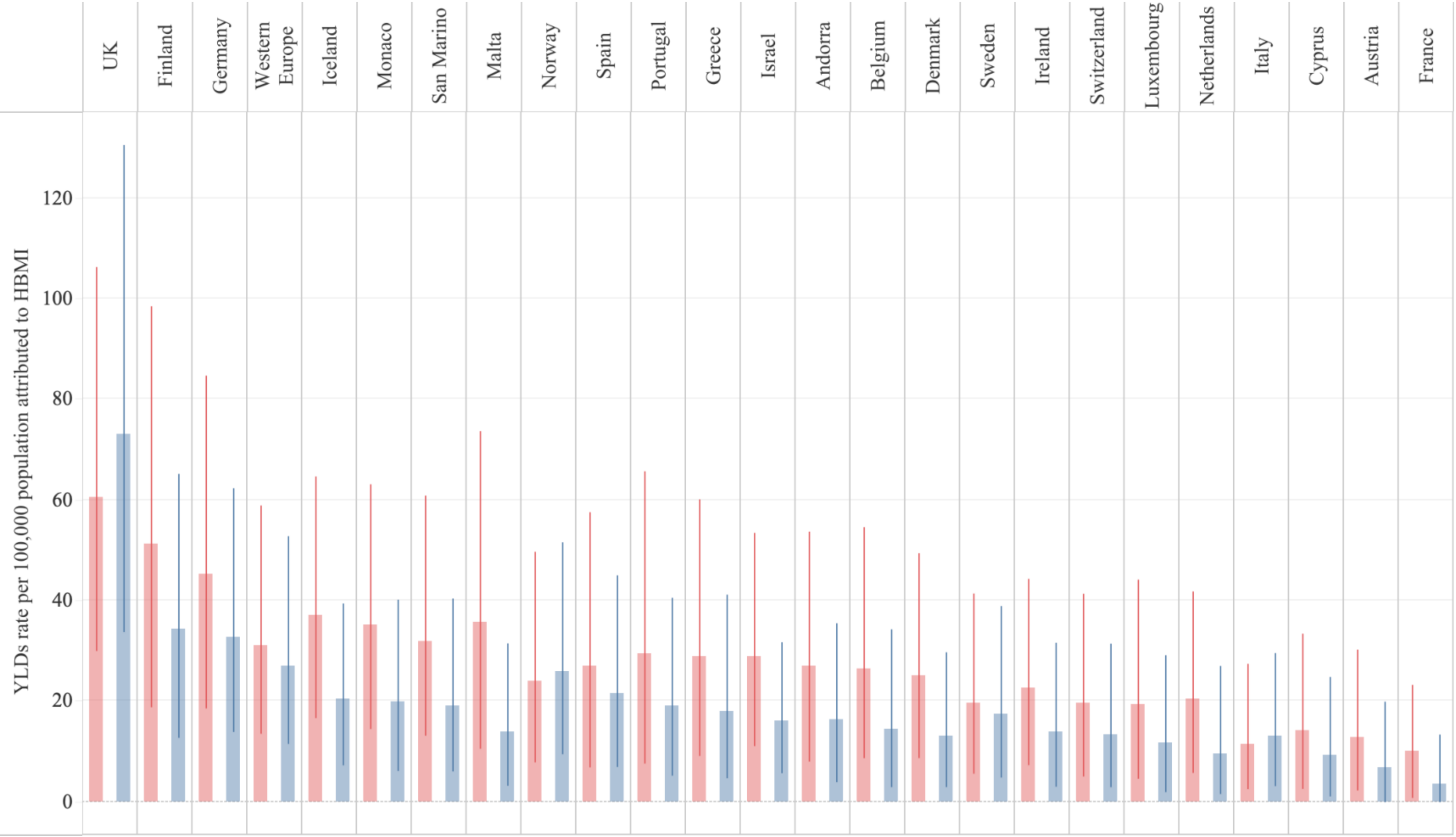

Abbreviations: T2DM=Type 2 diabetes mellitus; HBMI=High body mass index; UK=United Kingdom; 95% UI=95% uncertainty interval

Sex
Female
Male

Supplementary Figure 7. Summary exposure value rate per 100,000 attributed to HBMI in Western Europe, in 2019, by age groups (<24 years) and sex

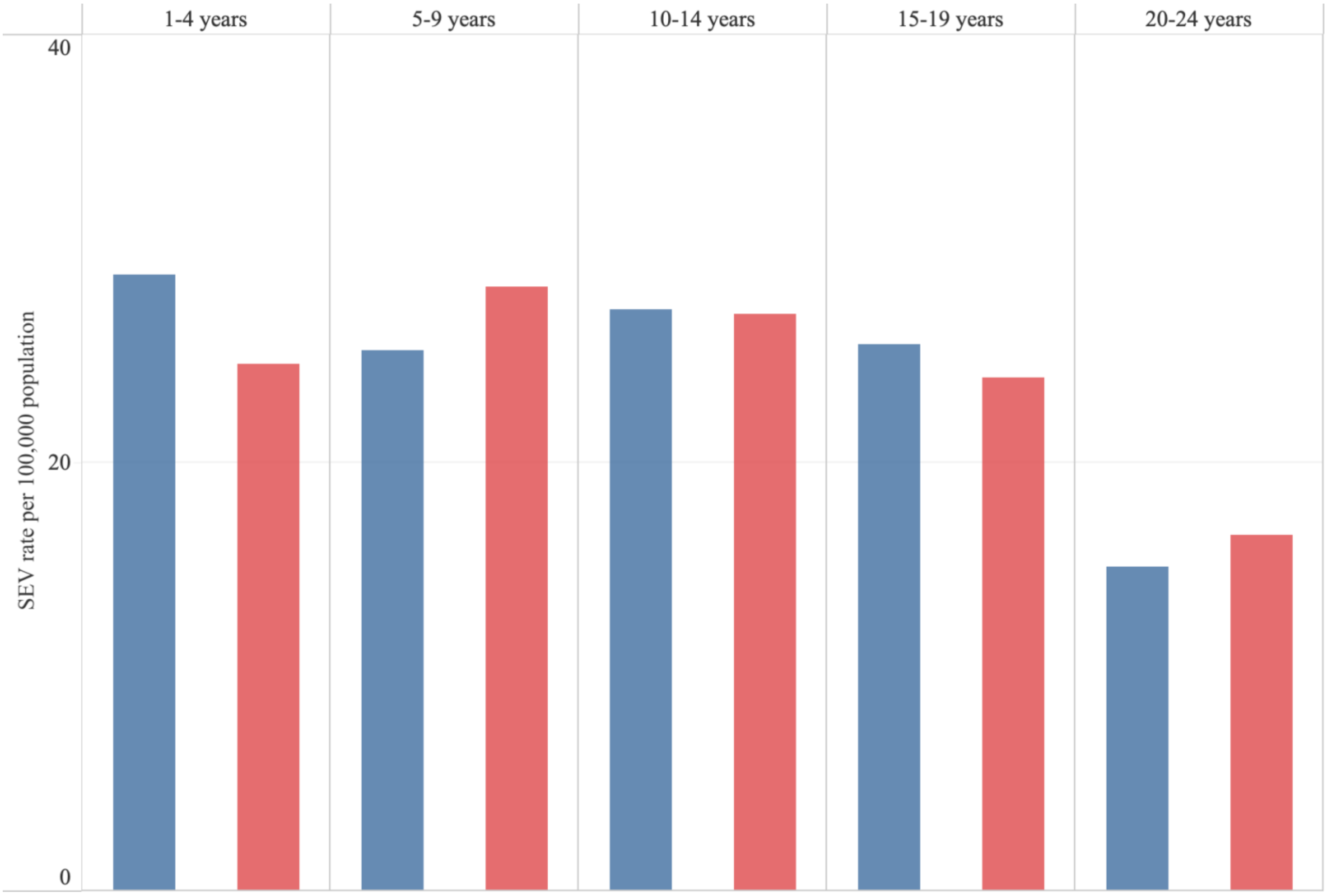

Abbreviations: HBMI=High body mass index; SEV=Summary exposure value

Sex
Female
Male

Supplementary Figure 8. Summary exposure value rate per 100,000 attributed to HBMI in Western Europe, by age groups (<24 years), sex and trend over time (1990-2019)

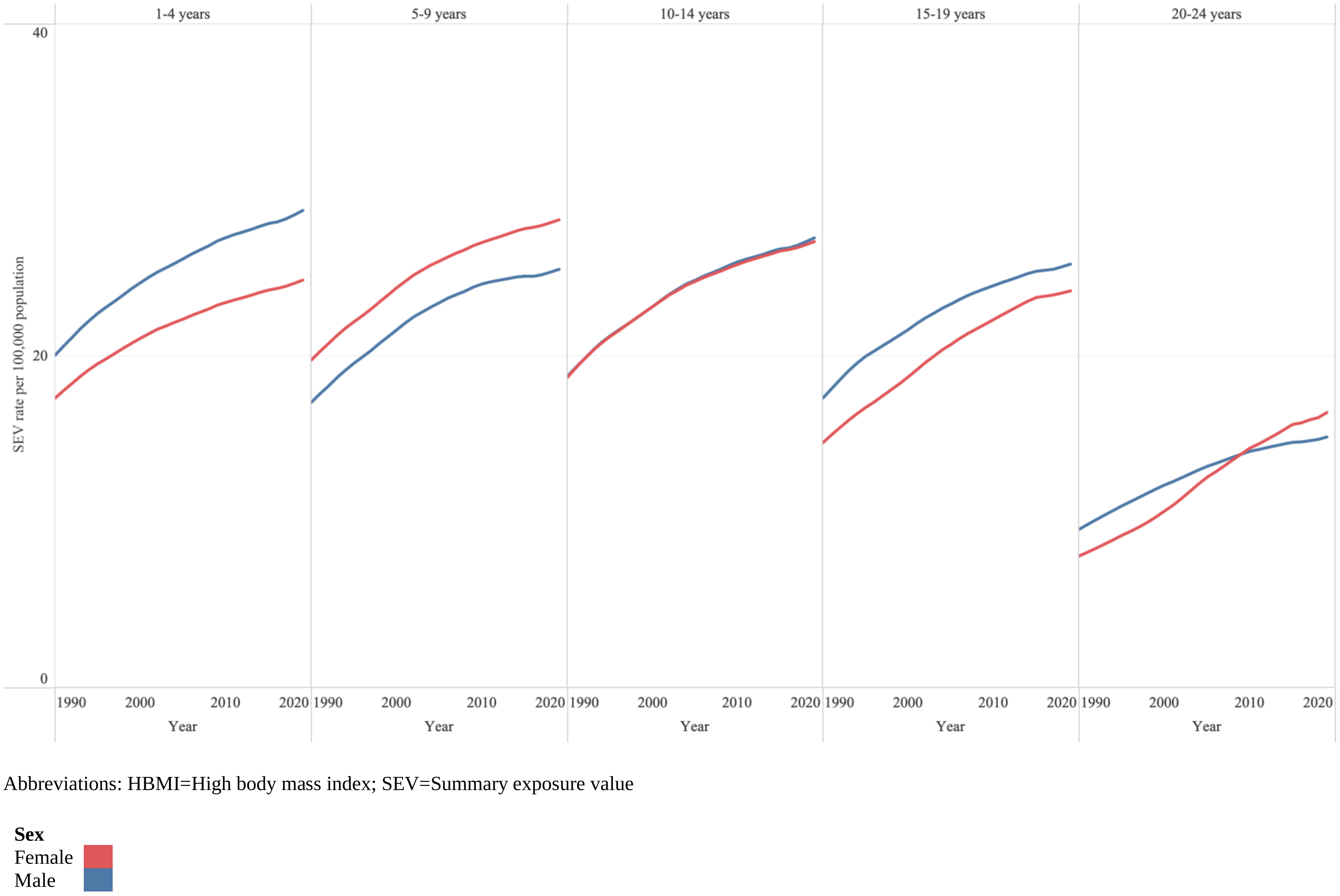

Abbreviations: HBMI=High body mass index; SEV=Summary exposure value

Supplementary Table 1. T1DM and T2DM prevalence, incidence and YLDs case numbers (with 95%UI), in 2019, in 24 Western European countries, by sex and age

Measure			Type 1 diabetes mellitus			Type 2 diabetes mellitus		
	Age (years)	Sex	Number	95%UI		Number	95%UI	
Prevalence	10-24	Male	167099	122432	212455	111128	63258	169598
		Female	153138	112031	195419	116171	66266	175536
		Both	320237	234473	408257	227299	130968	345987
	10-14	Male	39010	25794	52639	-	-	-
		Female	37110	25019	50081	-	-	-
		Both	76120	50886	102597	-	-	-
	15-19	Male	55481	39876	72004	15605	3920	33713
		Female	50980	36619	66562	17606	4553	36971
		Both	106461	76469	138376	33211	8397	70985
	20-24	Male	72607	55619	92170	95524	57260	140122
		Female	65048	49262	82846	98564	60656	141788
		Both	137655	104392	174656	194088	117970	280611
Incidence	10-24	Male	9646	6498	13300	33106	22762	46095
		Female	8155	5388	11334	29341	20371	40020
		Both	17800	11950	24643	62447	43141	86011
	10-14	Male	3682	1708	5426	-	-	-
		Female	3338	1524	4998	-	-	-
		Both	7020	3218	10414	-	-	-
	15-19	Male	2951	1566	4634	9528	5157	14468
		Female	2472	1297	3961	11278	6874	16146
		Both	5423	2872	8572	20806	12166	30537
	20-24	Male	3012	1217	5598	23578	15099	33619
		Female	2345	941	4358	18063	12324	25017
		Both	5358	2158	9977	41641	27484	58066
YLDs	10-24	Male	8435	5092	13086	5685	2728	10137
		Female	7786	4705	12036	6022	2925	10279
		Both	16222	9819	25029	11707	5642	20411
	10-14	Male	1912	1045	3062	-	-	-
		Female	1821	1004	2864	-	-	-
		Both	3733	2093	5896	-	-	-
	15-19	Male	2788	1695	4388	781	186	1891
		Female	2577	1539	4054	884	202	2032
		Both	5364	3253	8424	1664	402	3920
	20-24	Male	3736	2323	5695	4905	2391	8722
		Female	3388	2078	5119	5139	2615	8607
		Both	7124	4440	10823	10043	5095	17368

Abbreviation: YLDs=Years lived with disability; T1DM=Type 1 diabetes mellitus; T2DM=Type 2 diabetes mellitus; 95% UI=95% uncertainty interval
*Note: GBD study 2019 assumes that under 15 years all DM is T1DM

Supplementary Table 2. T1DM and T2DM prevalence, incidence and YLDs rate per 100,000 population (with 95%UI) in 2019, in 24 Western European countries, by sex and age

Measure			T1DM			T2DM		
	Age (years)	Sex	Rate	95%UI		Rate	95%UI	
Prevalence	10-24	Male	453.3	332.1	576.4	301.5	171.6	460.1
		Female	441.1	322.7	562.8	334.6	190.9	505.6
		Both	447.4	327.6	570.3	317.5	183.0	483.3
	10-14	Male	321.9	212.8	434.3	-	-	-
		Female	322.7	217.6	435.5	-	-	-
		Both	322.3	215.4	434.4	-	-	-
	15-19	Male	459.2	330.1	596.0	129.2	32.4	279.0
		Female	449.2	322.7	586.5	155.1	40.1	325.8
		Both	454.4	326.4	590.6	141.7	35.8	303.0
	20-24	Male	573.5	439.3	728.0	754.5	452.3	1106.7
		Female	547.9	415.0	697.8	830.2	510.9	1194.3
		Both	561.1	425.5	711.9	791.1	480.9	1143.8
Incidence	10-24	Male	26.2	17.6	36.1	89.8	61.8	125.0
		Female	23.5	15.5	32.6	84.5	58.7	115.3
		Both	24.9	16.7	34.4	87.2	60.3	120.2
	10-14	Male	30.4	14.1	44.8	-	-	-
		Female	29.0	13.3	43.5	-	-	-
		Both	29.7	13.6	44.1	-	-	-
	15-19	Male	24.4	13.0	38.4	78.9	42.7	119.8
		Female	21.8	11.4	34.9	99.4	60.6	142.3
		Both	23.1	12.3	36.6	88.8	51.9	130.3
	20-24	Male	23.8	9.6	44.2	186.2	119.3	265.5
		Female	19.8	7.9	36.7	152.1	103.8	210.7
		Both	21.8	8.8	40.7	169.7	112.0	236.7
YLDs	10-24	Male	22.9	13.8	35.5	15.4	7.4	27.5
		Female	22.4	13.6	34.7	17.3	8.4	29.6
		Both	22.7	13.7	35.0	16.4	7.9	28.5
	10-14	Male	15.8	8.6	25.3	-	-	-
		Female	15.8	8.7	24.9	-	-	-
		Both	15.8	8.9	25.0	-	-	-
	15-19	Male	23.1	14.0	36.3	6.5	1.5	15.7
		Female	22.7	13.6	35.7	7.8	1.8	17.9
		Both	22.9	13.9	36.0	7.1	1.7	16.7
	20-24	Male	29.5	18.3	45.0	38.7	18.9	68.9
		Female	28.5	17.5	43.1	43.3	22.0	72.5
		Both	29.0	18.1	44.1	40.9	20.8	70.8

Abbreviation: YLDs=Years lived with disability; T1DM=Type 1 diabetes mellitus; T2DM=Type 2 diabetes mellitus; 95% UI=95% uncertainty interval
*Note: GBD study 2019 assumes that under 15 all DM is T1DM

Supplementary Table 3. T1DM and T2DM incidence rate per 100,000 population (with 95%UI), in Western Europe and by 24 countries, in 1990 and 2019, with percentage change by time (with 95%UI)

Location	T1DM						Percentage change (1990-2019)			T2DM						Percentage change (1990-2019)		
	1990			2019						1990			2019					
	Rate	95%UI		Rate	95%UI		%	95%UI		Rate	95%UI		Rate	95%UI		%	95%UI	
Western Europe	14.3	11.1	17.8	24.9	16.7	34.4	75.5%	47.3%	100.5%	34.1	22.0	48.6	87.2	60.3	120.2	158.3%	134.6%	191.0%
Andorra	16.1	10.8	22.2	27.1	18.7	37.5	69.0%	46.1%	87.6%	27.6	16.0	40.4	67.5	44.9	94.8	146.6%	109.8%	197.9%
Austria	9.5	7.5	11.7	23.7	15.8	33.1	154.6%	88.5%	246.7%	23.4	15.4	33.2	49.3	31.6	70.8	115.0%	74.0%	159.2%
Belgium	11.9	8.5	15.5	23.0	15.3	32.1	94.9%	57.7%	133.9%	30.0	19.6	42.6	66.2	44.8	92.8	123.6%	90.0%	163.7%
Cyprus	10.9	8.1	13.8	25.0	16.7	34.5	131.4%	72.9%	216.9%	27.3	18.1	38.0	64.6	41.2	94.3	138.6%	97.6%	182.4%
Denmark	14.7	10.9	18.7	24.6	16.4	33.9	69.9%	36.2%	106.5%	29.5	19.0	42.4	64.9	44.9	88.6	122.2%	87.8%	169.3%
Finland	35.6	34.2	37.0	32.6	22.4	44.6	-7.5%	-35.8%	25.3%	47.0	28.8	70.1	114.7	81.5	154.6	146.2%	100.2%	223.6%
France	8.8	7.7	10.0	22.5	14.9	31.6	156.8%	79.4%	254.9%	15.6	10.3	21.9	36.0	22.1	53.1	131.2%	75.8%	188.5%
Germany	9.8	7.8	11.8	22.4	14.7	31.5	133.7%	63.1%	228.1%	49.2	34.5	67.2	105.6	75.2	144.6	118.2%	88.1%	151.6%
Greece	9.0	6.0	11.8	23.6	15.8	32.9	160.1%	94.4%	265.6%	28.5	19.1	39.6	75.0	50.2	104.8	161.8%	123.1%	207.9%
Iceland	13.3	8.7	19.5	20.0	12.9	28.3	51.6%	29.1%	72.0%	27.3	16.9	39.5	75.0	52.6	102.4	177.3%	141.0%	237.9%
Ireland	16.3	11.8	21.8	27.7	18.9	37.6	70.5%	37.6%	95.1%	18.9	10.4	28.1	53.1	34.1	74.1	182.1%	137.1%	264.5%
Israel	11.5	7.2	15.5	16.6	14.7	18.8	44.4%	9.4%	122.0%	24.8	16.7	34.8	61.1	43.7	81.1	147.1%	105.1%	209.7%
Italy	21.2	14.2	29.5	27.9	18.6	39.0	33.9%	19.4%	42.9%	35.1	18.4	54.8	56.7	32.7	85.3	64.0%	50.8%	90.6%
Luxembourg	12.7	8.9	16.5	23.9	15.9	33.3	92.6%	59.9%	126.3%	29.5	19.5	41.6	70.5	46.8	98.1	143.7%	113.1%	185.3%
Malta	17.6	11.8	24.4	27.5	18.6	38.2	56.6%	31.3%	77.2%	40.4	26.2	58.2	89.0	61.1	122.4	120.3%	88.2%	166.6%
Monaco	16.0	10.7	22.1	26.0	17.6	36.3	64.3%	44.3%	87.2%	29.8	17.2	44.3	72.3	47.9	100.8	145.0%	108.4%	203.2%
Netherlands	12.9	10.0	15.9	24.2	16.2	33.5	89.4%	48.2%	133.1%	26.6	16.9	38.3	57.2	37.1	80.6	116.5%	83.3%	163.6%
Norway	24.5	16.7	33.7	29.5	19.3	41.4	21.0%	11.6%	30.2%	51.7	31.8	74.4	73.0	48.0	102.4	42.2%	34.9%	54.8%
Portugal	13.0	9.2	17.0	26.2	17.7	36.1	106.5%	66.2%	162.6%	40.8	28.0	56.4	93.2	64.1	130.3	134.5%	101.6%	178.5%
San Marino	15.9	10.7	21.8	25.6	17.3	35.2	62.3%	42.0%	78.5%	28.6	17.1	42.6	72.3	48.1	99.3	153.8%	118.1%	211.6%
Spain	14.2	12.2	16.5	28.2	19.0	39.2	98.4%	41.7%	165.6%	29.2	19.3	41.6	78.5	53.0	109.4	168.5%	126.8%	222.5%
Sweden	24.8	16.7	34.1	27.3	18.1	38.2	9.8%	1.5%	18.1%	33.2	17.3	51.7	58.8	37.0	84.8	76.8%	49.1%	129.2%
Switzerland	16.1	10.8	22.3	26.5	18.0	36.6	68.0%	46.9%	85.9%	27.9	16.1	42.7	64.6	42.2	89.0	136.4%	103.4%	196.0%
UK	17.5	12.3	23.4	26.1	16.6	37.7	52.0%	29.5%	74.1%	43.1	25.7	63.3	180.2	129.2	243.0	326.2%	261.3%	449.3%

Abbreviation: T1DM=Type 1 diabetes mellitus; T2DM=Type 2 diabetes mellitus; 95% UI=95% uncertainty interval

Supplementary Table 4. YLDs rate per 100,000 for T2DM (with 95%UI), attributed to HBMI, in Western Europe and by 24 countries, in 1990 and 2019, with percentage change by time (with 95%UI)

Location	T2DM						Percentage of change		
	1990			2019					
	Rate	95%UI		Rate	95%UI			95%UI	
Western Europe	7.8	2.4	20.6	28.8	12.6	49.0	271.9%	180.6%	660.9%
Andorra	8.6	2.0	19.4	21.3	7.2	43.8	147.4%	56.9%	446.3%
Austria	3.1	0.4	5.5	9.7	1.4	17.3	211.3%	10.0%	578.3%
Belgium	6.4	1.4	7.7	20.3	6.6	23.1	216.4%	83.8%	1382.6%
Cyprus	4.2	0.8	10.2	11.6	2.3	28.1	178.8%	42.2%	617.2%
Denmark	5.6	1.1	26.6	18.9	6.5	72.6	240.3%	97.3%	207.4%
Finland	7.9	1.7	15.2	42.5	17.7	42.4	435.6%	218.3%	445.7%
France	2.1	0.4	17.0	6.6	0.4	54.9	211.6%	-55.3%	462.7%
Germany	12.5	3.7	14.1	38.5	17.8	39.1	207.1%	100.7%	560.2%
Greece	7.2	2.0	20.0	23.3	7.8	79.4	224.2%	103.6%	535.3%
Iceland	8.7	2.3	19.2	28.3	12.4	52.1	226.5%	114.9%	810.2%
Ireland	6.2	1.5	15.8	18.0	5.5	49.5	188.8%	62.8%	393.3%
Israel	5.3	1.0	13.7	22.2	9.4	36.7	315.9%	126.1%	730.3%
Italy	4.4	0.5	13.0	12.2	3.1	41.5	174.4%	92.0%	760.1%
Luxembourg	6.5	1.4	13.1	15.3	3.7	27.9	137.3%	34.1%	638.9%
Malta	10.8	2.6	10.9	24.4	7.7	32.6	127.1%	35.6%	1178.5%
Monaco	10.8	3.3	22.1	27.2	10.9	52.6	151.4%	70.8%	815.7%
Netherlands	4.2	0.8	17.4	14.8	4.1	39.3	254.1%	96.2%	589.2%
Norway	13.2	3.7	14.4	24.8	8.8	50.5	87.5%	47.8%	695.6%
Portugal	9.6	2.2	15.9	24.1	7.3	34.9	150.2%	59.7%	536.8%
San Marino	9.6	2.5	28.9	25.4	10.0	50.0	163.6%	72.4%	827.5%
Spain	6.3	1.6	26.1	24.2	8.0	51.1	285.2%	129.6%	356.4%
Sweden	6.6	1.0	14.1	18.5	5.4	35.5	180.4%	71.0%	467.5%
Switzerland	5.7	1.0	27.5	16.3	4.5	117.5	186.2%	73.0%	692.3%
UK	13.2	4.5	22.9	66.8	32.4	50.9	407.1%	276.6%	854.7%

Abbreviation: T2DM=Type 2 diabetes mellitus; 95% UI=95% uncertainty interval; HBMI=High body mass index
