# Supplementary material for: The Burden of Type 1 and Type 2 Diabetes Among Adolescents and Young Adults in 24 Western European Countries, 1990–2019: Results From the Global Burden of Disease Study 2019
Source: Int J Public Health. 2024 Feb 14;68:1606491. doi: 10.3389/ijph.2023.1606491 (PMC10899430; doi:10.3389/ijph.2023.1606491)
Supplement: Supplementary file 4 [file DataSheet2.docx]

## Authors’ Contributions

### Providing data or critical feedback on data sources

Hassan Abolhassani, Ayman Ahmed, Anton A Artamonov, Maciej Banach, Martina Barchitta, Till Winfried Bärnighausen, Boris Bikbov, Periklis Charalampous, Rajiv Chowdhury, Natália Cruz-Martins, Mostafa Dianatinasab, Peter Andras Gaal, Johannes Haubold, Claudiu Herteliu, Salman Hussain, Gaetano Isola, Mihajlo Jakovljevic, Adnan Kisa, Oleksii Korzh, Anders O Larsson, László Lorenzovici, Stefan Lorkowski, Andrea Maugeri, Tomasz Miazgowski, Irmina Maria Michalek, Ulrich Otto Mueller, Francesk Mulita, Christopher J L Murray, Gabriele Nagel, Ruxandra Irina Negoi, Bogdan Oancea, Adrian Pana, Maarten J Postma, Mónica Rodrigues, Milena M Santric-Milicevic, Rafael Tabarés-Seisdedos, Tommi Juhani Vasankari, Georgios-Ioannis Verras, Andrea Werdecker, Ronny Westerman, Mikhail Sergeevich Zastrozhin, and Magdalena Zielińska.

### Developing methods or computational machinery

Tareq Mohammed Ali AL-Ahdal, Mostafa Dianatinasab, Gaetano Isola, Adnan Kisa, Francesk Mulita, Christopher J L Murray, Michal Ordak, Mónica Rodrigues, and Ronny Westerman.

### Providing critical feedback on methods or results

Hassan Abolhassani, Tigist Demssew Adane, Ayman Ahmed, Catalina Liliana Andrei, Anton A Artamonov, Ashokan Arumugam, Muhammad Asaduzzaman, Maciej Banach, Martina Barchitta, Till Winfried Bärnighausen, Boris Bikbov, Aime Bonny, Periklis Charalampous, Rajiv Chowdhury, Natália Cruz-Martins, Omid Dadras, Mostafa Dianatinasab, Arkadiusz Marian Dziedzic, Florian Fischer, Peter Andras Gaal, Bishal Gyawali, Ahmad Hammoud, Johannes Haubold, Claudiu Herteliu, Salman Hussain, Irena M Ilic, Milena D Ilic, Gaetano Isola, Louis Jacob, Mihajlo Jakovljevic, Elham Jamshidi, Haitham Khatatbeh, Adnan Kisa, Oleksii Korzh, Ai Koyanagi, Carlo La Vecchia, Tea Lallukka, Jacopo Lenzi, László Lorenzovici, Stefan Lorkowski, Konstantinos Christos Makris, Hamid Reza Marateb, Andrea Maugeri, Tomislav Mestrovic, Junmei Miao Jonasson, Tomasz Miazgowski, Irmina Maria Michalek, Kebede Haile Misgina, Ulrich Otto Mueller, Francesk Mulita, Christopher J L Murray, Gabriele Nagel, Javaid Nauman, Ruxandra Irina Negoi, Bogdan Oancea, Michal Ordak, Alicia Padron-Monedero, Tamás Palicz, Adrian Pana, Ionela-Roxana Petcu, Vera Pinheiro, Maarten J Postma, Mónica Rodrigues, Milena M Santric-Milicevic, Art Schuermans, Rahman Shiri, Kerem Shuval, Luís Manuel Lopes Rodrigues Silva, Rafael Tabarés-Seisdedos, Seyyed Mohammad Tabatabaei, Georgios-Ioannis Verras, Isidora S Vujcic, Andrea Werdecker, Ronny Westerman, Mikhail Sergeevich Zastrozhin, and Magdalena Zielińska.

### Drafting the work or revising it critically for important intellectual content

Hassan Abolhassani, Ayman Ahmed, Catalina Liliana Andrei, Ashokan Arumugam, Muhammad Asaduzzaman, Maciej Banach, Martina Barchitta, Till Winfried Bärnighausen, Francesco Barone-Adesi, Luis Belo, Boris Bikbov, Nikolay Ivanovich Briko, Daniela Calina, Simiao Chen, Rajiv Chowdhury, Natália Cruz-Martins, Mostafa Dianatinasab, Monica Dinu, Arkadiusz Marian Dziedzic, Florian Fischer, Peter Andras Gaal, Bishal Gyawali, Ahmad Hammoud, Johannes Haubold, Claudiu Herteliu, Salman Hussain, Irena M Ilic, Milena D Ilic, Gaetano Isola, Louis Jacob, Mihajlo Jakovljevic, Haitham Khatatbeh, Adnan Kisa, Oleksii Korzh, Ai Koyanagi, Carlo La Vecchia, Tea Lallukka, Anders O Larsson, Jacopo Lenzi, László Lorenzovici, Stefan Lorkowski, Konstantinos Christos Makris, Hamid Reza Marateb, Andrea Maugeri, Tomislav Mestrovic, Tomasz Miazgowski, Irmina Maria Michalek, Kebede Haile Misgina, Ulrich Otto Mueller, Christopher J L Murray, Javaid Nauman, Ruxandra Irina Negoi, Bogdan Oancea, Ordak, Alicia Padron-Monedero, Tamás Palicz, Norberto Perico, Ionela-Roxana Petcu, Vera Pinheiro, Maarten J Postma, Giuseppe Remuzzi, Mónica Rodrigues, Milena M Santric-Milicevic, Art Schuermans, Kerem Shuval, Luís Manuel Lopes Rodrigues Silva, Johan Sundström, Rafael Tabarés-Seisdedos, Tommi Juhani Vasankari, Georgios-Ioannis Verras, Rade Vukovic, Andrea Werdecker, Ronny Westerman, Mikhail Sergeevich Zastrozhin, and Magdalena Zielińska.

### Managing the estimation or publications process

Gaetano Isola, Christopher J L Murray, and Mikhail Sergeevich Zastrozhin.
